# Inhibition of LRRK2 kinase activity rescues deficits in striatal dopamine physiology in VPS35 p.D620N knock-in mice

**DOI:** 10.1038/s41531-023-00609-7

**Published:** 2023-12-18

**Authors:** Mengfei Bu, Jordan Follett, Isaac Deng, Igor Tatarnikov, Shannon Wall, Dylan Guenther, Melissa Maczis, Genevieve Wimsatt, Austen Milnerwood, Mark S. Moehle, Habibeh Khoshbouei, Matthew J. Farrer

**Affiliations:** 1 Department of Neurology, University of Florida, Gainesville, FL USA; 2 McKnight Brain Institute, University of Florida, Gainesville, FL USA; 3 Djavad Mowafaghian Centre for Brain Health, University of British Columbia, Vancouver, BC Canada; 4 Department of Neurology & Neurosurgery, Montreal Neurological Institute, McGill University, Montreal, QC Canada; 5 Department of Pharmacology and Therapeutics, University of Florida, Gainesville, FL USA; 6 Department of Neuroscience, University of Florida, Gainesville, FL USA

**Keywords:** Parkinson's disease, Neurophysiology, Transporters in the nervous system

## Abstract

Dysregulation of dopamine neurotransmission profoundly affects motor, motivation and learning behaviors, and can be observed during the prodromal phase of Parkinson’s disease (PD). However, the mechanism underlying these pathophysiological changes remains to be elucidated. Mutations in vacuolar protein sorting 35 (*VPS35*) and leucine-rich repeat kinase 2 (*LRRK2*) both lead to autosomal dominant PD, and VPS35 and LRRK2 may physically interact to govern the trafficking of synaptic cargos within the endo-lysosomal network in a kinase-dependent manner. To better understand the functional role of VPS35 and LRRK2 on dopamine physiology, we examined Vps35 haploinsufficient (Haplo) and Vps35 p.D620N knock-in (VKI) mice and how their behavior, dopamine kinetics and biochemistry are influenced by LRRK2 kinase inhibitors. We found Vps35 p.D620N significantly elevates LRRK2-mediated phosphorylation of Rab10, Rab12 and Rab29. In contrast, Vps35 haploinsufficiency reduces phosphorylation of Rab12. While striatal dopamine transporter (DAT) expression and function is similarly impaired in both VKI and Haplo mice, that physiology is normalized in VKI by treatment with the LRRK2 kinase inhibitor, MLi-2. As a corollary, VKI animals show a significant increase in amphetamine induced hyperlocomotion, compared to Haplo mice, that is also abolished by MLi-2. Taken together, these data show Vps35 p.D620N confers a gain-of-function with respect to LRRK2 kinase activity, and that VPS35 and LRRK2 functionally interact to regulate DAT function and striatal dopamine transmission.

## Introduction

Parkinson’s disease (PD) is the most common aged-related movement disorder. Motor symptoms are associated with the insidious but progressive loss of striatal dopaminergic innervation that ascends from neuronal soma in the midbrain. Most patients with late-onset PD are idiopathic and have a multifactorial etiology. This includes a well-defined background of polygenic risk, albeit of marginal effect^1^, and rare patients can have a causal monogenic component^2^. Dominantly-inherited mutations in leucine-rich repeat kinase 2 (*LRRK2* p.N1437H, p.R1441C/G/H, p.Y1699C, p.G2019S and p.I2020T)^3–6^ and vacuolar protein sorting 35 (*VPS35* p.D620N)^7,8^ have been genetically linked to parkinsonism that is clinically and pathologically indistinguishable from idiopathic PD^2^. These mutations are some of the most penetrant causes of PD and confer high genotypic risk.

VPS35 is an essential component of the retromer complex, responsible for endosomal sorting and recycling of cargo proteins, that precludes their endo-lysosomal degradation^9^. *VPS35* knock out is embryonic lethal, and its deficiency impairs mitochondrial fusion and induces age-dependent α-synuclein pathology^10–12^. In vitro studies of Vps35 p.D620N have shown the mutation neither affects protein expression nor disturbs retromer complex assembly^13^. However, many studies have found the amino acid substitution compromises cargo sorting functions of the retromer complex, consistent with a partial loss-of-function^13–16^. Previously we showed Vps35 p.D620N knock-in mice (VKI) have early alterations to the nigrostriatal dopamine system^17^. Subsequent studies demonstrate VKI mice exhibit age-dependent tau pathology and dopaminergic neuronal loss^18,19^. Nevertheless, the pathophysiological role of VPS35 in the dopaminergic system remains ill-defined and whether the p.D620N mutation confers a gain- or loss-of-function is unclear^15^.

Pathogenic mutations in *LRRK2* increase its kinase activity, directly^20^ and indirectly^21–23^. Brain imaging in clinically asymptomatic *LRRK2* heterozygotes reveals differences in their dopaminergic and serotonergic systems^24^. LRRK2 substrate phosphorylation is also significantly elevated in neutrophils and monocytes from heterozygous patients with *VPS35* p.D620N parkinsonism^25^, and in fibroblasts and brain tissue obtained from VKI mice^14,25^. Multiple studies have shown that VPS35 and LRRK2 physically and functionally interact to regulate vesicular trafficking^14,16,25–27^. Overexpression of *Vps35* may rescue locomotor deficits in Drosophila expressing mutant *Lrrk2*^28^ but is toxic in mammalian neuronal cultures and greatly diminishes synaptic activity and density^16,29^. The 1:1:1 stoichiometry of VPS35:VPS29:VPS26 subunits (the retromer ‘core’ trimer) needs to be maintained so results from non-physiologic overexpression of a single subunit must be interpreted cautiously^13^. The basic biology and interactions between LRRK2 and VPS35 warrant further investigation in the dopaminergic system, given the therapeutic potential of these targets to PD.

To assess gain- versus loss-of-function of Vps35 p.D620N, and to better understand the relationship between VPS35 and LRRK2, we performed parallel investigations in Vps35 p.D620N (VKI) and Vps35 haploinsufficient (Haplo) mice (in which Vps35 expression is reduced by 50%). Outcome measures focused on LRRK2 substrate phosphorylation in VPS35 models, and how LRRK2 kinase inhibitors influence their biochemistry, behavior and dopamine kinetics. We demonstrate LRRK2 substrates are differentially phosphorylated in VKI and Haplo animals, and find amphetamine-induced hyperlocomotion in WT and VKI mice is impaired in Haplo mice. Most remarkably, LRRK2 kinase inhibition reduces amphetamine-induced hyperlocomotion in VKI mice, while normalizing DAT expression and function. Together, the data reveal distinct differences between WT, VKI, and Haplo mice within the nigrostriatal dopamine system consistent with a LRRK2-dependent gain-of-function in VPS35 p.D620N parkinsonism.

## Results

### LRRK2 kinase activity is differentially affected by Vps35 haploinsufficiency and the Vps35 p.D620N mutation

**Fig. 1:**
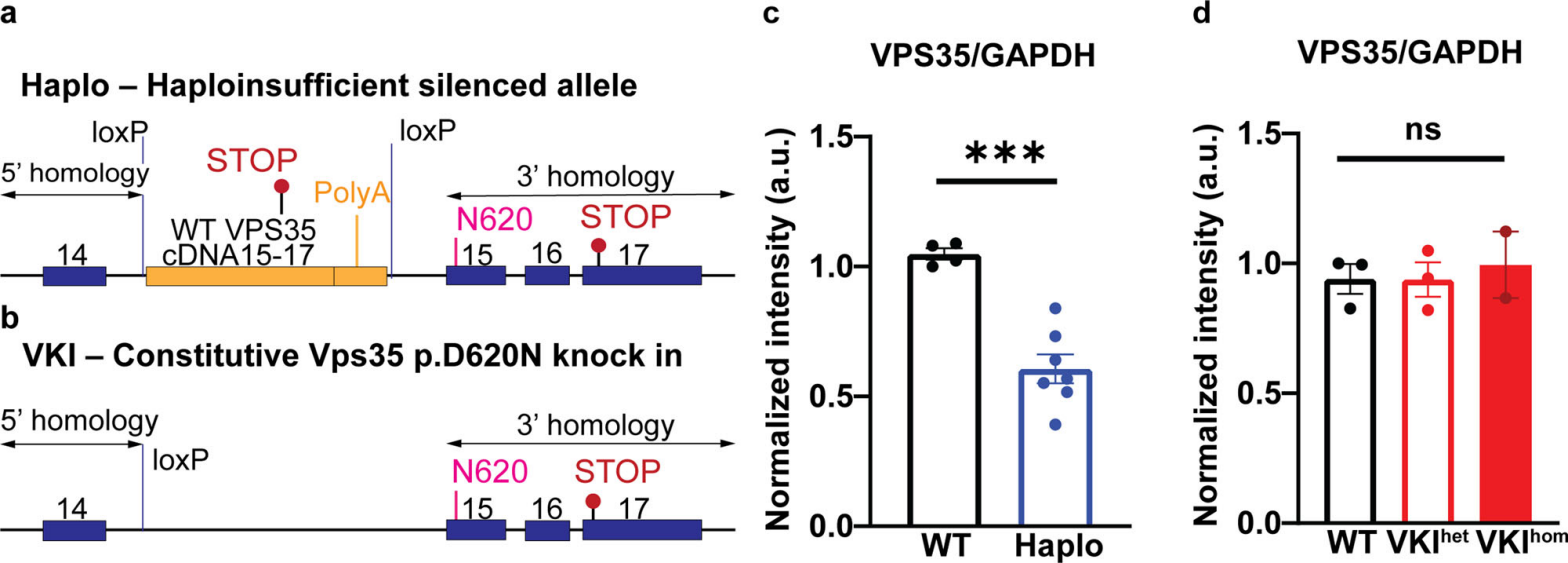
Generation of Vps35 haploinsufficient (Haplo) and Vps35 p.D620N knock-in (VKI) mice. Schematics of targeting design for (**a**) Haplo animals showing the murine Vps35 genomic sequence (Ensembl reference ENSMUSG00000031696), 5′ and 3′ homology arms (arrowed), exons 14–17 (blue), engineered loxP site in intron 14, the g.85,263,520 G > A mutation in exon 15 (encoding the p.N620 substitution, in pink) and endogenous stop codon in exon 17 (TAA, lollipop in red). A cDNA cassette of wild type murine ‘exons 15–17 with a polyA tail’ (yellow) was originally introduced to create a conditional knock-in (VKI) but inadvertently silenced the endogenous allele. **b** VKI animals created by Cre-recombinase deletion between loxP sites. Total VPS35 protein level was quantified by western blotting, showing 50% reduction in Haplo compared to their WT littermates (**c**: WT: *N* = 4, Haplo: *N* = 7; unpaired *t* test, t_9_ = 5.76, p < 0.001) and no difference in Het and Hom VKI (**d**: WT: *N* = 3, Het: *N* = 3, Hom: *N* = 2; One-way ANOVA, F_2, 5_ = 0.15, *p* = 0.87). *p < 0.05, **p < 0.01, ***p < 0.001. Error bars are ±SEM.

We generated Vps35 haploinsufficient mice (Haplo) and Vps35 knock-in mice (VKI) that constitutively express the Vps35 p.D620N mutation, genetically linked to PD^7^ (Materials and Methods, Fig. 1a, b). Haplo animals exhibit a 50% reduction in Vps35 expression (Fig. 1c), whereas hetero- and homozygous VKI animals exhibit comparable expression of Vps35 compared to wild type (WT) littermates (Fig. 1d)^17^. Both Haplo and VKI animals are viable and fertile, whereas homozygous Vps35 knockout is embryonic lethal^30^. Haplo animals are a bona-fide loss of Vps35 function model. Thus, we performed a comparative examination of VKI and Haplo animals at 3-months of age to investigate gain- versus loss-of-function of VPS35 with respect to LRRK2 biology, and to assess the interplay of VPS35-LRRK2 in the dopamine system.

**Fig. 2:**
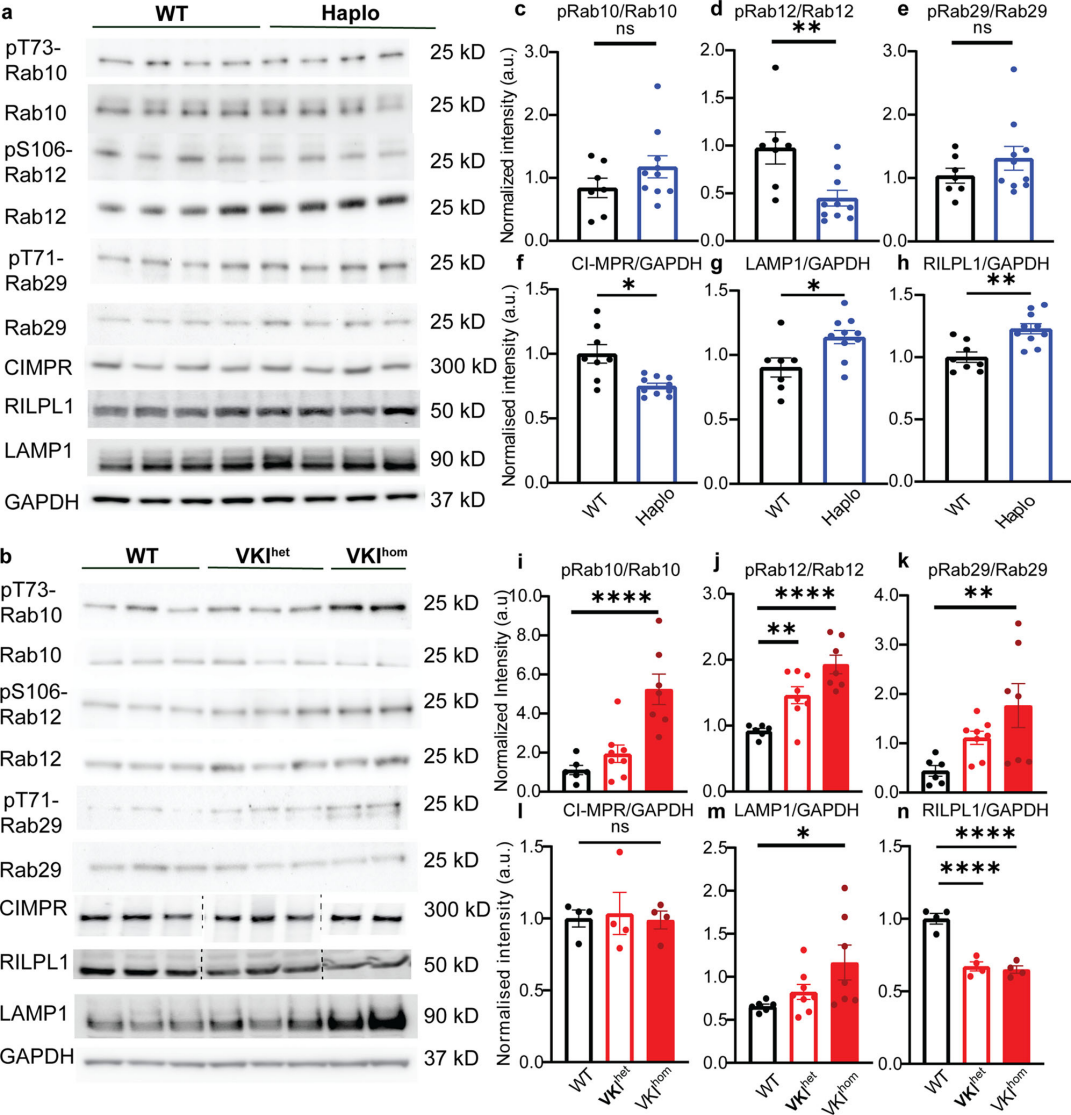
Rab protein phosphorylation is reduced in Haplo but increased in VKI. Representative blots of striatal lysate of Haplo (**a**) and VKI (**b**) animals respectively. **c–n** Quantifications of immunoblots: Haplo animals exhibit comparable phosphorylation of Rab10 (**c**) and Rab29 (**d**) but reduced Rab12 phosphorylation (**e**). (**c–e**: WT: *N* = 7, Haplo *N* = 10, unpaired *t* test **c**: t_15_ = 1.35; **d**: t_15_ = 3.34; **e:** t_15_ = 1.05). Haplo animals exhibit reduced CI-MPR (**f** unpaired *t* test with Welch’s correction, t_8.32_ = 3.29), and increased LAMP1 (**g: WT:** ***N*** = **7, Haplo** ***N*** = 10, unpaired *t* test, t_15_ **=** **2.69**) and RILPL1 (**h**: unpaired *t* test, t_16_ = 3.87)(**f, h**: WT: *N* = 8, Haplo *N* = 11). **i–k** VKI exhibit a significant increase in Rab10, 12, 29 phosphorylation (**i–k:** WT: *N* = 6, VKI^het^: *N* = 8, VKI^hom^: *N* = 7; One-way ANOVA with Dunnett’s multiple comparison test, (**i**) F_2, 18_ = 18.29, (**j**): F_2, 18_ = 25.06; (**k**): F_2, 18_ = 5.22). VKI exhibit a comparable level of CI-MPR (**l**: WT: *N* = 4, VKI^het^: *N* = 4, VKI^hom^: *N* = 4; One-way ANOVA F_2, 9_ = 0.058), increased LAMP1(**m**: WT: *N* = 6, VKI^het^: *N* = 8, VKI^hom^: *N* = 7; One-way ANOVA with Dunnett’s multiple comparison test, F_2, 18_ = 3.70) and reduced RILPL1. (**n:** WT: *N* = 4 animals; VKI^het^: *N* = 4 animals; VKI^hom^: *N* = 4 animals; One-way ANOVA with Dunnett’s multiple comparison F_2, 9_ = 39.01). Note that full blots and quantifications of CI-MPR and RILPL1 in VKI homogenates are shown in Supplementary Fig. 2. *p < 0.05, **p < 0.01, ***p < 0.001, ***p < 0.0001. Error bars are ±SEM.

We first performed a cursory examination of the endo-lysosomal pathway by western blot analysis of striatal homogenates from VKI and Haplo mice. We used Rab10 (pThr73), Rab12 (pSer106) and Rab29 (pThr71) as indirect readouts of LRRK2 kinase activity and as markers for endosomal, lysosomal and Golgi trafficking, respectively^25,31–33^. We found Haplo animals exhibit comparable levels of pRab10/Rab10 (Fig. 2c) and pRab29/Rab29 (Fig. 2e) but had a significant reduction in pRab12/Rab12 (Fig. 2d) compared to WT littermates. Cation-independent mannose-phosphate receptor (CI-MPR), a classic retromer cargo, is reduced in Haplo confirming a loss of VPS35 function (Fig. 2f), but was unchanged in VKI (Fig. 2l). Previous work has shown LRRK2 kinase activation in VKI^25^. Here, we confirmed an increase in pRab10/Rab10 and pRab12/Rab12 and showed that pRab29/Rab29 is also significantly increased in striatal homogenates from 3-month-old VKIs (Fig. 2i–k). Treatment with MLi-2 in vivo (7-day intraperitoneal injection, 5 mg/kg dose), a potent LRRK2 kinase inhibitor, significantly abolished Rab phosphorylation in VKIs with a clear interaction between treatment and genotype, further supporting that hyperphosphorylation of Rab proteins in VKIs are LRRK2 kinase dependent (Supplementary Fig. 1). Upon examining down stream lysosomal and autophagic markers, we observed a significant increase in lysosomal associated protein-1 (LAMP1) in both models (Fig. 2g, m) as well as changes in several other autophagic markers (Supplementary Fig. 2). Rab Interacting Lysosomal Protein Like 1 (RILPL1) is significantly increased in Haplo but reduced in VKI (Fig. 2h, n). RILPL1 is recruited to lysosomes upon lysosomal stress in a LRRK2 kinase-dependent manner, resulting in accelerated degradation and reduced RILPL1 expression in whole cell lysate^34^. Together, our data suggests that retromer loss-of-function alone is not sufficient to drive LRRK2 activation, and the Vps35 p.D620N mutation may selectively impair trafficking of specific cargos. While both Vps35 haploinsufficiency and the p.D620N mutation may lead to endo-lysosomal deficits, our data shows p.D620N induces a gain-of-function with respect to LRRK2 kinase activity.

### Vps35 haploinsufficiency or p.D620N mutant dysfunction has minimal effect on basal dopaminergic neuron pace-making activity

**Fig. 3:**
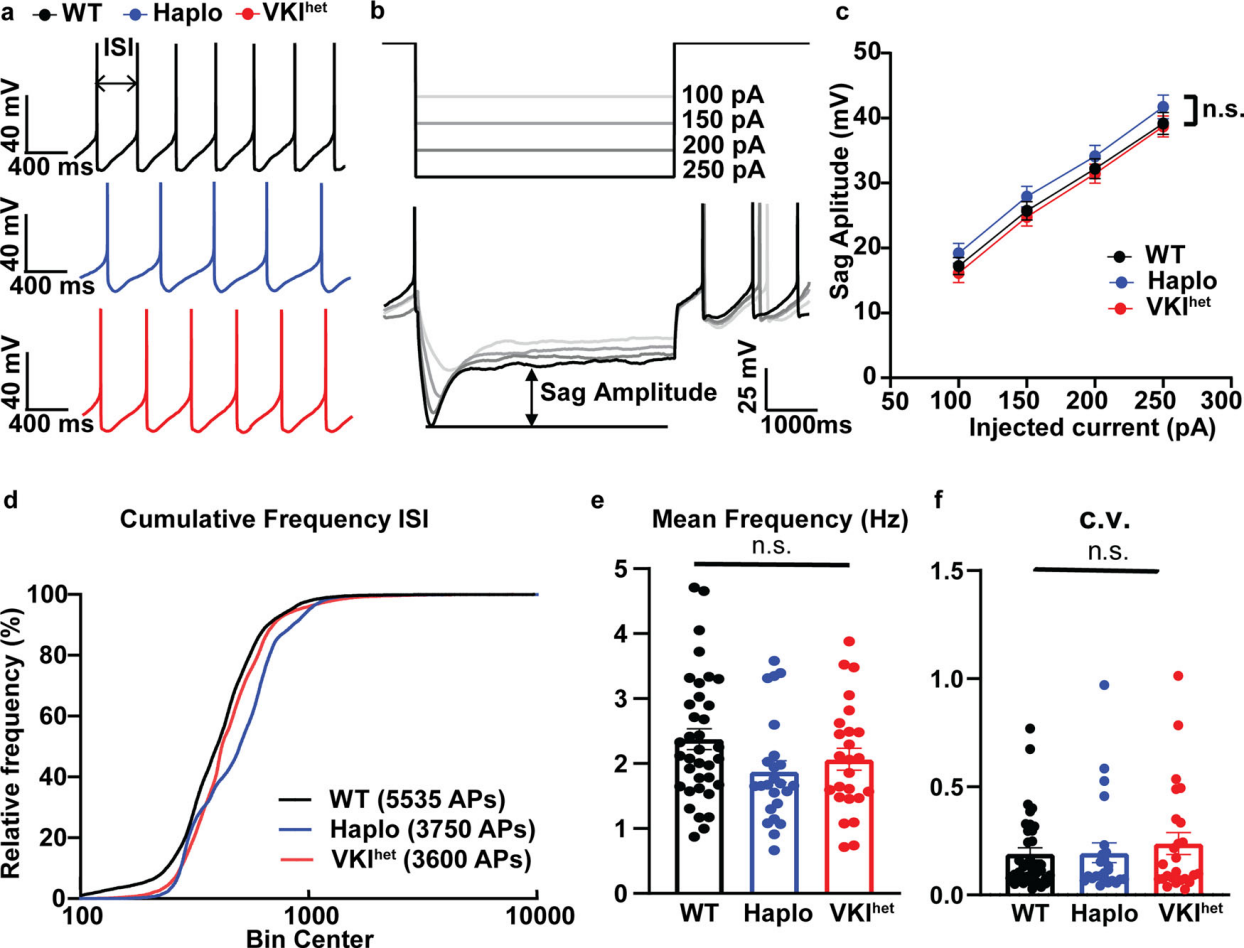
Haplo and VKI SNc dopaminergic neurons exhibit comparable basal pace-making activity. **a** Representative traces of perforated, spontaneously active SNpc dopaminergic neurons. **b** Representative traces displaying depolarizing sag potentials in response to hyperpolarizing current injections in SNpc dopaminergic neurons. **c** Summary plot of sag amplitude shows comparable I_H_ activation in VKI^het^ and Haplo (Two-way ANOVA; WT: *n* = 34, *N* = 7, VKI^het^ *n* = 25, *N* = 5, Haplo: *n* = 24, *N* = 5; genotype: F_2, 78_ = 0.96, *p* > 0.05; current: F _1.637, 127.7_ = 704.3, *p* < 0.0001; Genotype x current: F_6, 234_ = 0.087, *p* > 0.99 Subject: F_78, 234_ = 23.01, *p* < 0.0001). **d** Cumulative frequency plot of inter-spike intervals (ISIs) of all the action potentials (APs) shows comparable ISIs in Haplo compared to WT and VKI^het^. (WT: *n* = 5535 APs, Haplo: *n* = 3750 APs, VKI^het^: *n* = 3600 APs, One-way ANOVA F _2, 720_ = 1.41, *p* > 0.05). **e**, **f** VKI^het^ or Haplo exhibit no significant difference in firing frequency (**d**: WT: *n* = 34 cells, *N* = 7 animals, VKI *n* = 25 cells, *N* = 5 animals, Haplo: *n* = 24 cells, *N* = 5 animals; One-way ANOVA, F_2, 82_ = 2.37, *p* = 0.10) or the fidelity of the pace-making activity as reflected by coefficient of variance (C.V.) of all ISIs. (**f**: WT: *n* = 34 cells, *N* = 7 animals, VKI *n* = 25 cells, *N* = 5 animals, Haplo: *n* = 24 cells, *N* = 5 animals, Kruskal-Wallis test, *p* = 0.82). *p < 0.05, **p < 0.01, ***p < 0.001. Error bars are ±SEM.

The loss of dopaminergic neurons in the *substantia nigra pars compacta* (SNpc) leads to the cardinal motor symptoms of PD. To examine how dysregulation of VPS35 affects SNpc dopaminergic neurotransmission, we used ex-vivo perforated patch clamp electrophysiology to examine autonomous pace-making activity, an energy-demanding process that may contribute to the selective vulnerability of these neurons^35,36^. SNpc dopaminergic neurons are identified based on their large cell body, their spontaneous firing (1–5 Hz), broad action potentials (APs) > 1.5 ms (Fig. 3a), and prominent depolarizing sag during hyperpolarizing current injection (Fig. 3b)^37^. The sag potential in SNpc dopaminergic neurons is regulated by hyperpolarization-activated cyclic nucleotide-gated (HCN, I_h_) cation channels, an important regulator in pace-making activity^37,38^. Both Haplo and VKI^het^ dopamine neurons exhibit comparable levels of sag potential reflecting normal HCN function (Fig. 3c). Firing frequency (Fig. 3d, e) and the fidelity (Fig. 3f) of pace-making activity are also comparable among dopaminergic neurons of WT, Haplo and VKI^het^. Together, these results suggest that the pace-making activity of SNpc dopaminergic neurons is intact in 3-month-old Haplo and VKI^het^ animals.

### Haplo and VKI animals exhibit similar dysfunction in striatal dopamine release

**Fig. 4:**
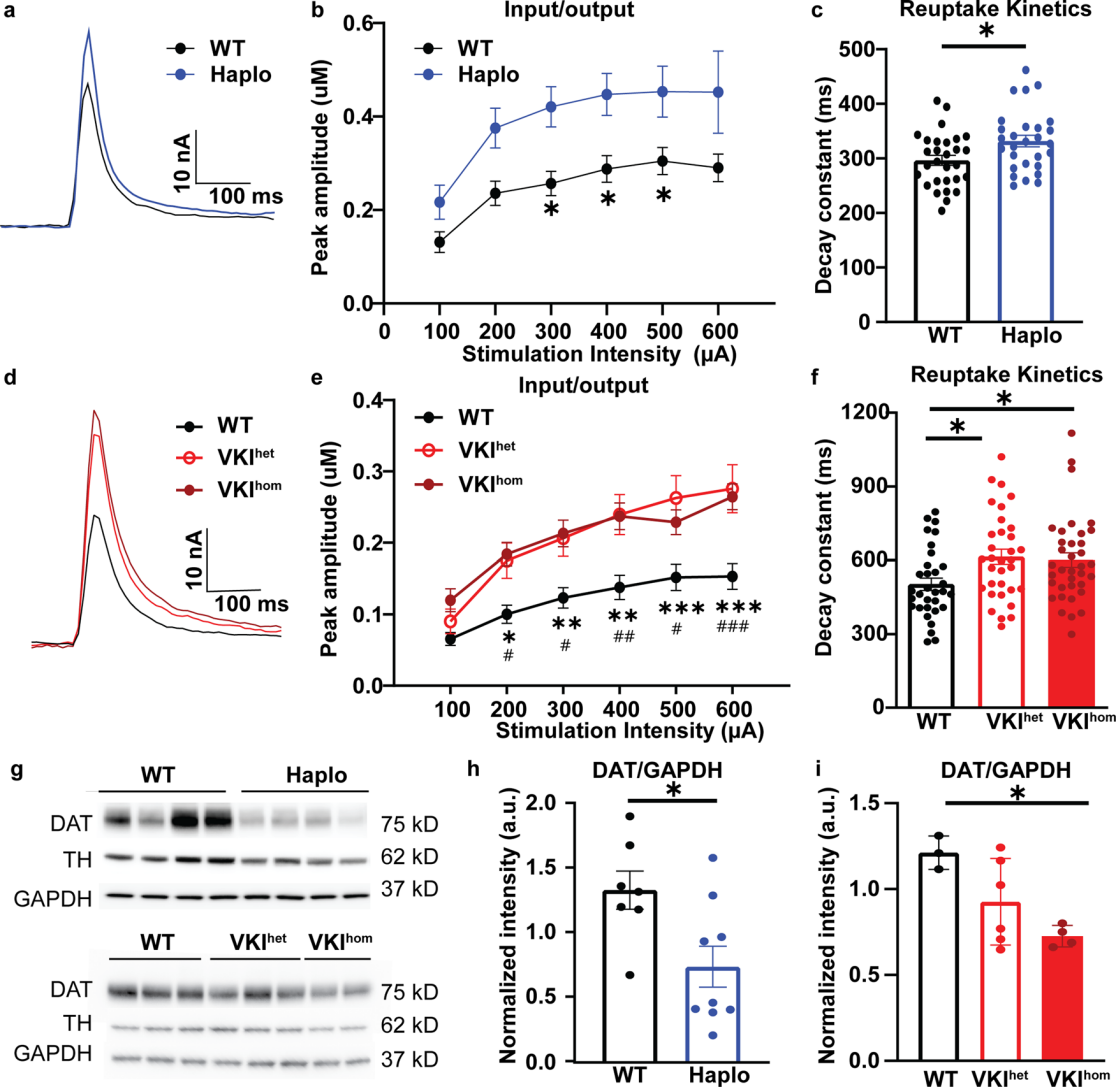
Haplo and VKI mice exhibit increased evoked peak amplitude, reduced DA reuptake and reduced DAT expression. **a**, **d** Representative traces of evoked dopamine response following single pulse stimulation in Haplo and VKI animals respectively. Haplo slices exhibit significant increase in evoked peak amplitude as reflected by input/output curves (**b**: WT *n* = 29 slices. *N* = 6; Haplo *n* = 28 slices, *N* = 5 animals; Mixed effects ANOVA with Sidak’s multiple comparisons test, stimulation intensity: F_5, 253_ = 28.33, *p* < 0.0001; genotype: F_1, 55_ = 8.20, *p* < 0.01; stimulation intensity x genotype F_5, 253_ = 1.02, *p* = 0.41. WT v.s. Haplo: *p < 0.05, **p < 0.01, ***p < 0.001) and prolonged dopamine reuptake kinetics as reflected by increased decay constant τ (**c**: WT *n* = 29 slices. *N* = 6; Haplo *n* = 28 slices, *N* = 5 animals; unpaired *t* test, t_55_ = 2.53, *p* < 0.05). **e–f** VKI exhibit a significant increase in evoked peak amplitude as reflected by input/output curves (**e**: Mixed effects ANOVA with Sidak’s multiple comparisons test, stimulation intensity: F_6, 588_ = 87.54, *p* < 0.0001; genotype: F_2, 98_ = 7.10, *p* < 0.05; stimulation intensity x genotype F_12, 588_ 3.87, *p* < 0.0001; Bonferroni post-test WT vs VKI^het^ *p < 0.05, **p < 0.01, ***p < 0.001, WT vs VKI^hom^; #*p* < 0.05, ##*p* < 0.01, ###*p* < 0.001) and prolonged dopamine reuptake kinetics as reflected by the increased decay constant τ (**f**: One-way ANOVA F_2, 98_ = 4.37, *p* < 0.02, Dunnett’s multiple comparison test, t_98_ = 2.71, *p* < 0.05, and t_98_ = 2.42, *p* < 0.05 for VKI^het^ and VKI^het^ slices). (**d–f** adapted from Caltaldi et al., 2018). **g** Representative western blots of dopaminergic markers in striatal tissue. Quantification of western blots shows a significant reduction in total striatal DAT expression in Haplo (**h**, WT: *N* = 7, Haplo: *N* = 10; unpaired *t* test; t_15_ = 2.62, *p* < 0.05) and VKI mice (**i**, WT *N* = 3, VKI^het^: *N* = 5, VKI^hom^: *N* = 5; One-way ANOVA, F_2, 10_ = 9.53, *p* < 0.01; Dunnett’s multiple comparisons test; WT vs. VKI^het^ *p* = 0.13, WT vs. VKI^hom^, *p* < 0.01). Data normalized to GAPDH loading control. *p < 0.05, **p < 0.01, ***p < 0.001. Error bars are ±SEM.

SNpc dopaminergic neurons project extensively to the dorsal lateral striatum. Dopamine release at striatal axonal terminals critically regulates sensorimotor behavior and activity, independent of the pace-making of soma in the midbrain^39–41^. To examine the effects of VPS35 dysfunction in striatal dopamine dynamics, we performed ex-vivo fast scan cyclic voltammetry in acute striatal slices of Haplo and VKI animals. Upon electric stimulation, Haplo slices exhibit elevated peak amplitude in dopamine release across a broad range of stimulation intensities (Fig. 4a, b), and a significant increase in the decay constant tau (Fig. 4c), reflecting prolonged dopamine reuptake. This response phenocopied age-matched striatal slices of VKI, which also exhibit enhanced peak amplitude and prolonged reuptake kinetics (Fig. 4d–f). Dopamine concentration in the dorsal striatum is dependent on the dopamine transporter (DAT). Notably, the prominent DAT blocker, nomifesine, significantly increased peak evoked dopamine release in all genotypes and to a comparable level (Supplementary Fig. 3a–c). Haplo and VKI mice have a significant reduction in striatal DAT, without a significant difference in mRNA (Fig. 4g, Supplementary Fig. 3h). Collectively, these results are consistent with previous literature showing DAT as a retromer cargo^42^ and provide complementary in-vivo evidence that dysregulation of retromer may impair DAT trafficking, which in turn leads to dysregulation of striatal dopamine dynamics.

### Haplo and VKI animals exhibit divergent locomotion responses to amphetamine

**Fig. 5:**
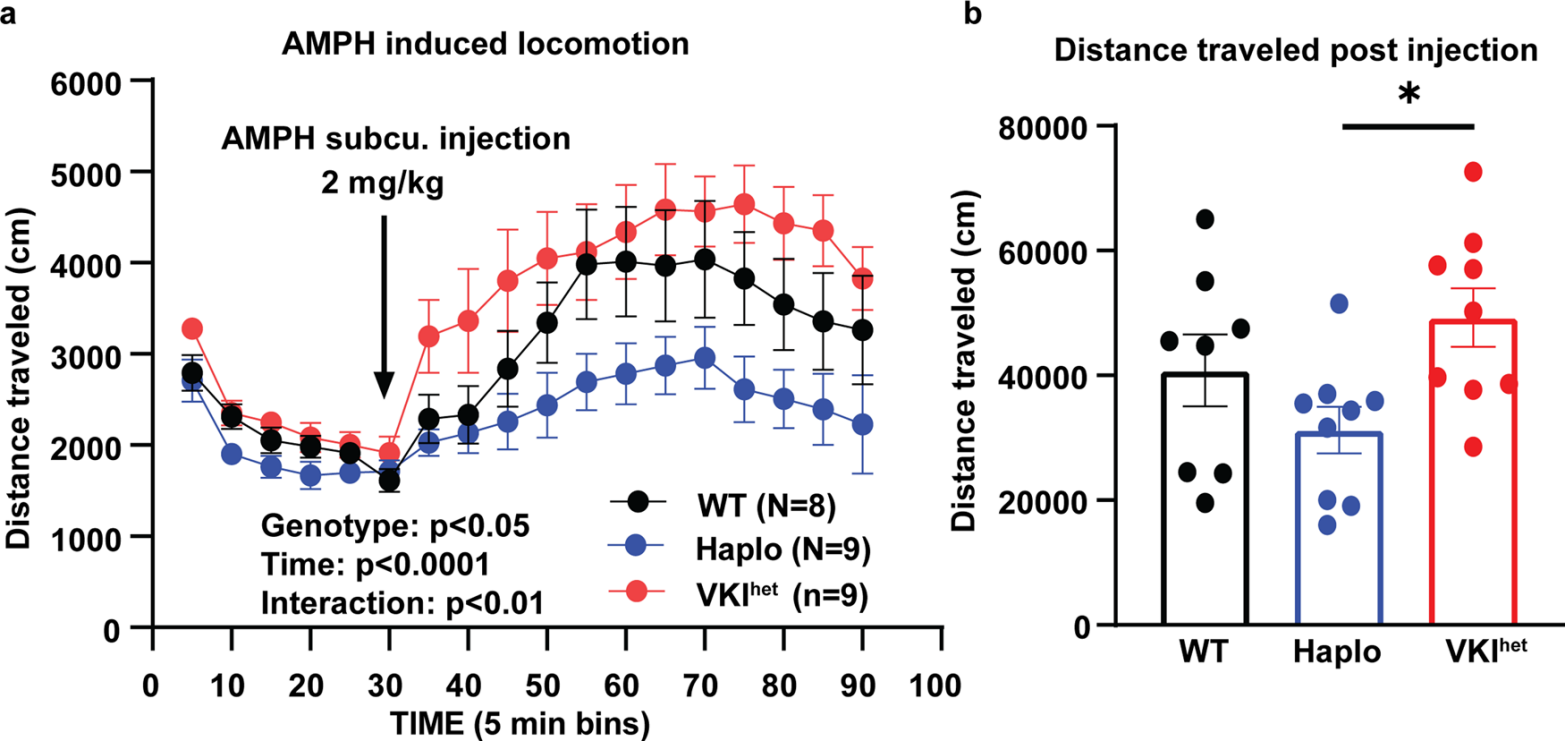
Haplo and VKI exhibit divergent responses to amphetamine-induced locomotion. **a** Distance traveled over 90 min of open field monitoring. Haplo and VKI^het^ animals exhibit divergent responses to AMPH (WT: *N* = 8 animals, Haplo *N* = 9 animals, VKI^het^ *N* = 9 animals; Two-way ANOVA: Genotype: F_2, 23_ = 5.04, *p* < 0.05; Time: F_2.483, 57.11_ = 22.41, *P* < 0.0001; Time x Genotype F_34, 391_ = 1.76, *p* < 0.01; Subject: F_23, 391_ = 20.78, *p* < 0.0001). **b** Total distance traveled post injection. (WT: *N* = 8 animals, Haplo *N* = 9 animals, VKI^het^: *N* = 9 animals; One-way ANOVA; F_2, 22_ = 5.14, *p* < 0.05; Tukey’s multiple comparison test Haplo vs. VKI^het^: *p* < 0.05). *p < 0.05, **p < 0.01, ***p < 0.001. Error bars are ±SEM.

Nigro-striatal dopamine neurotransmission critically regulates locomotion. We and others have previously characterized motor function in Haplo and VKI mice at 3 months of age, but found no significant differences in open field exploration, rotarod or cylinder test at baseline^11,17,30^. Given the constitutive nature of our genetic model, it is possible that the neuronal network has adapted to subtle changes in dopamine transmission which could mask behavioral responses. Therefore, we acutely challenged the dopamine system with amphetamine (AMPH), a potent stimulator of dopamine release via DAT reverse transport and vesicular depletion, and measured AMPH-induced locomotion in Haplo and VKI^het^ mice. Animals were placed in an open field chamber for 30 min prior to subcutaneous injection of AMPH at 2 mg/kg, and then monitored for an additional 60 min within the open field chamber. AMPH-induced hyperlocomotion in WT, VKI^het^ and Haplo; however, Haplo mice exhibited a reduced response compared to WT and VKI^het^ mice (Fig. 5a, b) consistent with the effects of AMPH treatment in DAT knockout mice^43,44^. In contrast, although VKI^het^ mice also have reduced DAT reuptake they exhibit a rapid and robust increase in their locomotion, and initially exceeding that observed in WT. Hence, Vps35 p.D620N confers a gain-of-function to maintain AMPH-induced locomotion.

### Pharmacological inhibition of Lrrk2 kinase activity in-vivo rescues dopaminergic phenotypes in VKI

**Fig. 6:**
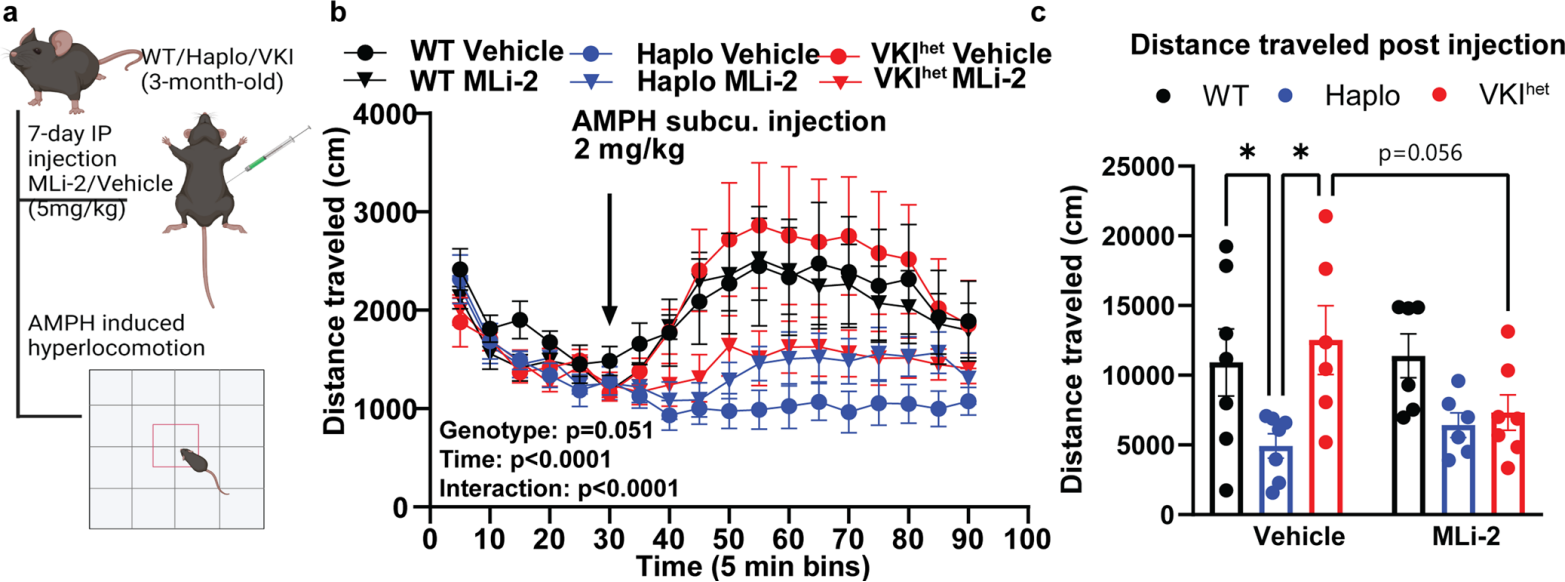
7-day in-vivo treatment by MLi-2 reduces AMPH-induced hyperlocomotion in VKI to levels comparable to Haplo mice. **a** Schematic of MLi-2 treatment and experimental paradigm (Created with BioRender). **b** Distance traveled over 90 min of open field monitoring (WT vehicle, *N* = 7, WT MLi-2: *N* = 7, Haplo vehicle: *N* = 7, Haplo MLi-2: *N* = 6, VKI^het^ vehicle: *N* = 6, VKI^het^ MLi-2: *N* = 7; Two-way ANOVA: Group: F _5, 33_ = 2.48, *p* = 0.051; Time: F _2.053, 67.76_ = 9.33, *p* < 0.001; Time x Group: F _85, 561_ = 2.115, *p* < 0.0001; Subject F_33, 561_ = 22.78, *p* < 0.0001). **c** Total distance traveled post injection. (WT vehicle, *N* = 7, WT MLi2: *N* = 7, Haplo vehicle: *N* = 7, Haplo MLi-2: *N* = 6, VKI^het^ vehicle: *N* = 6, VKI^het^ MLi-2: *N* = 7; Two-way ANOVA: Genotype: F _(2, 33)_ = 5.653, *p* < 0.05; Treatment F _1, 33_ = 0.59, *p* = 0.44; Genotype x Treatment F_2, 33_ = 2.22, *p* = 0.12; Sidak multiple comparisons *p < 0.05, **p < 0.01, ***p < 0.001). Error bars are ±SEM.

Next, we investigated whether the divergent responses in AMPH-induced hyperlocomotion in Haplo and VKI^het^ are dependent on LRRK2 activity. We acutely inhibited LRRK2 kinase activity in-vivo using MLi-2, a potent LRRK2 kinase inhibitor. 3-month-old animals received either vehicle or MLi-2 treatment for 7 days through intraperitoneal injection (Fig. 6a, Supplementary Fig. 2). Interestingly, while MLi-2 treatment has minimal effects on WT and Haplo mice, it attenuated AMPH-induced hyperlocomotion in VKI^het^ animals to levels comparable to Haplo (Fig. 6b, c) suggesting that AMPH-induced hyperlocomotion in VKI^het^ is dependent on LRRK2 kinase activation.

**Fig. 7:**
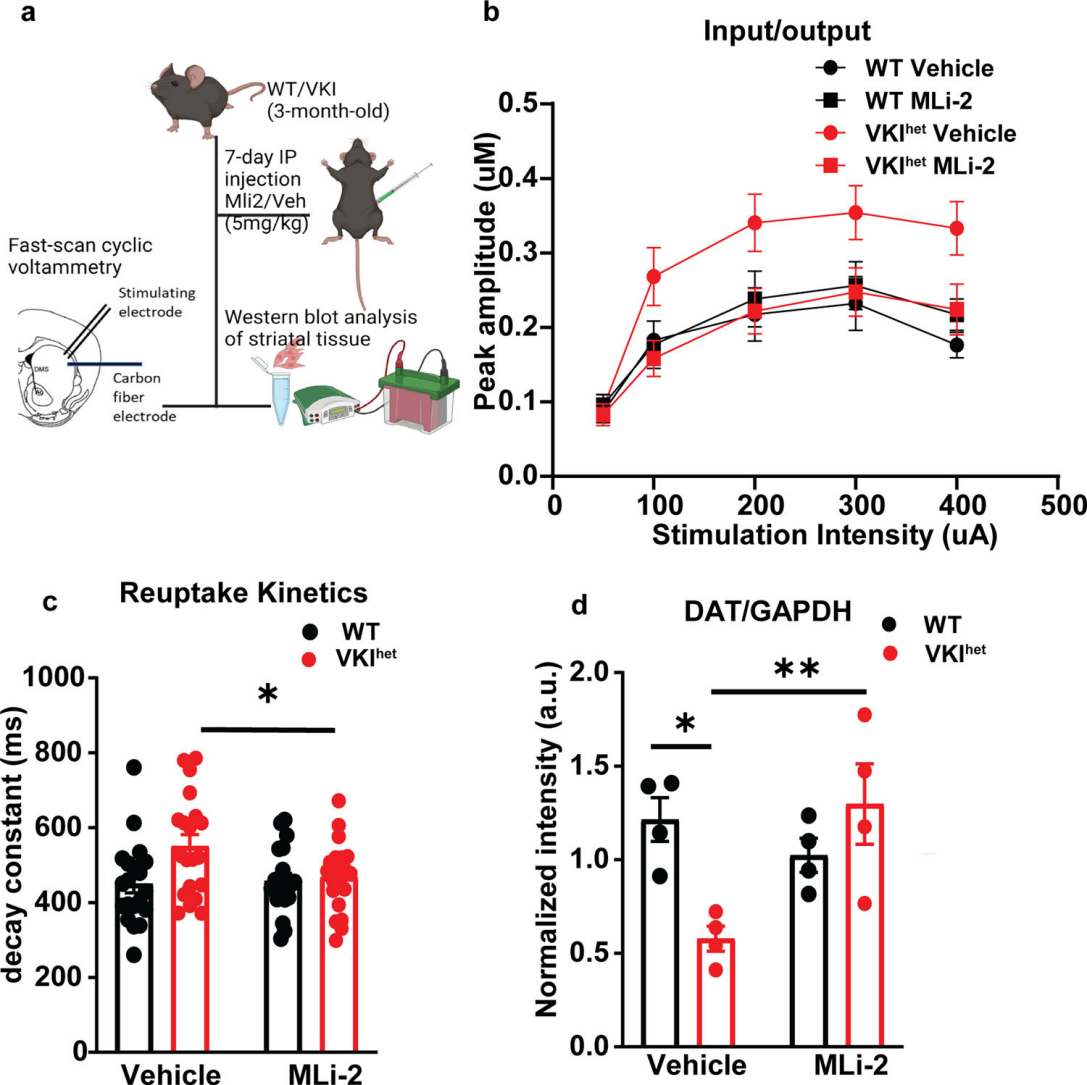
7-day in-vivo treatment by MLi-2 normalizes evoked striatal dopamine response and Dat expression in VKI. **a** Schematic of MLi-2 treatment and experimental paradigm (Created with BioRender). **b** MLi-2 treatment normalizes input/output response of VKI^het^ to levels comparable to WT (WT vehicle: *n* = 19 slices, *N* = 5 animals, WT MLi-2: *n* = 20 slices, *N* = 5 animals, VKI^het^ vehicle: *n* = 20 slices, *N* = 5 animals, VKI^het^ MLi-2: *n* = 20 slices, *N* = 5 animals; Mixed effect analysis: stimulation intensity: F_4, 184_ = 21.99, *p* < 0.0001; Genotype: F_1, 184_ = 9.89, *p* < 0.01; Treatment: F_1, 161_ = 7.57, *p* < 0.01; Genotype x Treatment: F_1, 161_ = 16.57, *p* < 0.0001). **c** MLi-2 treatment normalizes reuptake kinetics of VKI^het^ to level comparable to WT. (WT vehicle: n = 19 slices, *N* = 5 animals, WT MLi-2: *n* = 20 slices, *N* = 5 animals, VKI^het^ vehicle: *n* = 20 slices, *N* = 5 animals, VKI^het^ MLi-2: *n* = 20 slices, *N* = 5 animals; Two-Way ANOVA: Genotype, F_1, 76_ = 5.33, *p* < 0.05; Treatment, F_1, 76_ = 2.40, *p* = 0.13; Genotype x Treatment: F_1, 76_ = 3.49, *p* = 0.066; Tukey’s multiple comparisons: VKI^het^ vehicle vs VKI^het^ MLi-2: *p* < 0.05). **d** MLi-2 treatment normalizes DAT expression level in VKI^het^. (*N* = 4 for all groups; Two-Way ANOVA: Genotype, F_1, 12_ = 1.81, *p* = 0.20; Treatment: F_1, 12_ = 3.84, *p* = 0.074; Genotype x Treatment: F _1, 12_ = 11.39, *p* < 0.01; Tukey’s multiple comparisons: VKI^het^ vehicle vs VKI^het^ MLi-2: *p* < 0.05; VKI^het^ vehicle vs. WT vehicle: *p* < 0.05) *p < 0.05, **p < 0.01, ***p < 0.001. Error bars are ±SEM.

Phosphorylation locks Rab proteins in their GTP-bound forms, prevents their interaction with effector proteins and stalls vesicular trafficking. To test whether LRRK2 mediated hyperphosphorylation of Rab proteins impairs DAT trafficking in VKI^het^, we re-examined evoked striatal dopamine dynamics in VKI^het^ mice treated with vehicle or MLi-2 (Fig. 7a). Remarkably, MLi-2 treatment normalized dopamine dynamics in VKI^het^ to levels comparable to WT, both in terms of peak amplitude (Fig. 7b) and reuptake kinetics (Fig. 7c). At the protein level, the initial reduction in striatal DAT expression in VKI^het^ animals was also raised to levels comparable to WT (Fig. 7d). Taken together, these results suggest that LRRK2 functions downstream of VPS35 to regulate DAT trafficking and dopamine signaling.

## Discussion

Deficits in endo-lysosomal trafficking are associated with PD-related genes and may disproportionally affect dopaminergic neurons due to their extensively arborized axons and requirement for nigrostriatal axonal trafficking^45,46^. Here we show that Vps35 p.D620N confers a gain-of-function with respect to LRRK2 kinase activity, and a reduction of DAT function and expression. Moreover, acute inhibition of LRRK2 kinase activity is sufficient to rescue these deficits in nigrostriatal dopamine dynamics and related AMPH-induced behavior. A strength of our work is that we are able to directly compare Haplo and VKI results, as these mouse models were derived from the same founder and have been maintained on the same genetic background (Fig. 1). By focusing on DAT, a known retromer cargo specific to dopaminergic terminals, we were able to assess the interplay between VPS35 and LRRK2 in a cell specific manner in vivo. Our approach was designed to be etiologically faithful and physiologically relevant to dopamine biology, which is clearly compromised in PD. When examining the effect of LRRK2 inhibition, we focused on VKI^het^ as heterozygous animals are efficient to generate and homozygous human subjects with VPS35 p.D620N have never been described. Hence, results in the VKI^het^ model and human subjects with VPS35 p.620N may inform the development of LRRK2 kinase inhibitors as a disease-modifying therapy.

VPS35 and LRRK2 functionally interact to regulate multiple vesicular trafficking pathways including endocytic trafficking^14,47^, synaptic vesicle recycling^27^ and trans-Golgi network (TGN) trafficking^48^. By western blotting, we identified a significant increase in the phosphorylation of Rab proteins, notably pRab10, pRab12 and pRab29 in VKI compared to WT, suggesting Rab-mediated endosomal, lysosomal and Golgi trafficking may be compromised. Owing to the difficulty in detecting pS1292 LRRK2 in murine brain under basal conditions, we were not able to directly observe a significant increase in this epitope. Nevertheless, application of the LRRK2 kinase inhibitor MLi-2 significantly reduced pRabs in VKI mice with a clear interaction between treatment and genotype, suggesting Rab hyper-phosphorylation in VKI mice is LRRK2 kinase mediated (Fig. 2). In contrast, there is little evidence for dysregulation of LRRK2 kinase activity in Vps35 haploinsufficient animals, since we observed a significant reduction in pRab12/Rab12 and no changes in pRab10/Rab10 or pRab29/Rab29 (Fig. 2). The reduction in pRab12/Rab12 may reflect a compensatory increase in endo-lysosomal flux as retromer cargos are inadvertently retained, rather than efficiently recycled^49–51^. Hence, in agreement with others, we provide in-vivo evidence that Vps35 p.D620N results in a gain-of-function with respect to LRRK2 kinase activity^25^. Nevertheless, protein phosphorylation is also temporally labile and is more challenging to quantify from dissected tissues than cell lysates, and both these phenomena may lead to disparities in the literature. For example, we note that siRNA knockdown of Vps35 in mouse embryonic fibroblasts induces a significant reduction in pRab10/total Rab10 signal^25^.

At 3-months of age, we observed no significant change to the pace-making activity of the dopaminergic soma in the SNpc (Fig. 3) but evoked striatal dopamine release is significantly enhanced in Haplo and VKI slices, along with a significant reduction in DAT expression and function (Fig. 4). Dopaminergic terminals in the striatum are under the control of local micro-circuitry^41,52^ and striatal dopamine release can be decoupled from the firing activity of the soma^39,40^. Vps35 is enriched at dopaminergic release sites^53^ thus it is not surprising to see that Vps35 dysfunction can lead to dysregulated dopaminergic neurotransmission in a region-specific manner. We found both Vps35 haploinsufficiency and the p.D620N mutation impairs DAT function and expression. This complements previous studies that show DAT is a cargo protein for retromer-dependent trafficking^42,54^. The effect of VPS35 p.D620N on DAT and dopamine dynamics is sex-specific, as FSCV in female VKI^het^ mice are comparable to WT (unpublished data), and warrants a more systematic evaluation of female VKI and Haplo mice.

The behavioral response of Haplo mice to AMPH was blunted (Fig. 5) similar to hetero- and homozygous DAT knockout mice^43,44^. The lack of effect of MLi-2 in Haplo mice may be a floor effect; nevertheless, WT animals respond more than Haplo at baseline and still show no change in AMPH-induced locomotion after MLi-2 treatment (Fig. 6). Despite deficits in DAT, VKI^het^ exhibit enhanced AMPH-induced locomotion compared to Haplo, which is abolished by MLi-2 treatment (Figs. 5–6). Hence, Vps35 p.D620N likely confers a gain-of-function to maintain AMPH locomotion that is LRRK2 kinase-dependent. However, the exact pathway through which LRRK2 acts to maintain hyperlocomotion in VKI warrants further study. One possible contribution is changes in dopamine metabolism. We previously examined dopamine release in VKI in-vivo, using microdialysis and HPLC, and found a significant increase in dopamine metabolites (homovanillic acid (HVA) and 3,4-dihydroxyphenylacetic acid (DOPAC)) without any change in dopamine^17^, suggesting a potential increase in monoamine-oxidase (MOA) activity. Notably, MOA is inhibited by AMPH which may further elevate cytosolic and extracellular dopamine contributing to the increased locomotion observed^55^. Alternatively, VKIs could compensate to maintain AMPH-induced locomotion through VMAT2, as AMPH depletes dopamine vesicular storage through dissipation of proton gradients in VMAT2+ vesicles^55^. With immunoblotting we observed an increased in VMAT2 expression in VKI^het^ in drug naïve mice (Supplementary Fig. 4). However, even with a good antibody, VMAT2’s function can not be informed by immunoblotting alone. Future imaging studies with either fluorescent-false neurotransmitter or ^3^[H] dihydrotetrabenzine binding may help illuminate on the role of VPS35 and LRRK2 on VMAT2 function^56–59^. Other than affecting the mesolimbic dopamine system, AMPH also promotes norepinephrine and serotonin release via VMAT2, norepinephrine (NET) and serotonin transporters (SERT) throughout the brain, albeit with limited contribution to locomotion^60^. In addition, VKI mice may exhibit dysregulation in downstream circuits that are also dependent on LRRK2, which is more abundant in medium spiny neurons, innervating cortical synapses^61,62^ and glia^63^ than dopaminergic terminals or axons^64–66^. Many consider the function of LRRK2 to be as, if not more, important in immune cells including microglia. Hence, future studies should explore the relationship between VPS35, recycling retromer cargos and LRRK2 in different cellular contexts.

DAT critically regulates dopamine neurotransmission and its function is dynamically regulated by multiple endocytic trafficking pathways^67^. A reduction in DAT density and increased dopamine turnover are observed in early symptomatic and pre-symptomatic individuals with monogenic mutations causal for parkinsonism^64,65,68^. Thus, early alterations in striatal dopamine dynamics and DAT expression in Haplo and VKI mice are consistent with human imaging results. Although there is no overt neurodegeneration at 3-month of age^17^, VKI mice do exhibit an age-dependent loss of midbrain dopaminergic neurons^19^, a cardinal feature of PD. Collectively, our results and prior studies highlight VKI mice as a biologically and clinically relevant model to study molecular and circuit mechanisms underlying the dysfunction of dopamine neurons before the onset of neurodegeneration.

Intriguingly, acute inhibition of LRRK2 activity also rescued striatal DAT expression and dopamine dynamics in ex-vivo slice preparations (Fig. 7). It’s unlikely the results of our 7-day treatment paradigm are dependent on the synthesis of new DAT. The level of DAT transcripts in both models are comparable (Supplementary Fig. 3). In-situ hybridization of DAT mRNA shows an intense signal in the midbrain and is minimal in striatum (https://mouse.brain-map.org/gene/show/12942)^66^, suggesting DAT transcriptional and translational production in the striatum is below detection threshold. Rather, DAT synthesis occurs in the soma; subsequent anterograde axonal transport is mainly through membrane diffusion, with only a minor contribution from vesicular trafficking, and it takes around 20 days for new DAT to replenish levels in the striatum^54^. In dopaminergic terminals, DAT may be trafficked and possibly degraded through conventional endocytic trafficking mechanisms, rather than by autophagy, although more localization studies and consensus is needed^69–71^. However, several technical limitations must be overcome to study DAT colocalization and protein interactions in a physiologic context. Here we postulate LRRK2 kinase inhibition in VKI mice may rescue DAT through three mechanisms: (1) via endosomal retrieval and recycling in a Rab-dependent manner^72–75^; (2) through RIT2, a neural GTPase implicated in sex- and regionally-specific DAT trafficking^76,77^, and/or; (3) Rho GTPase and actin cytoskeletal rearrangements^78^, in which the role of LRRK2 kinase activity must be explored.

For the first time, we demonstrate a functional interaction between VPS35 and LRRK2 in dopaminergic neurons, in-vivo, and in the control of locomotion. These neurons preferentially die in PD. In VKI mice, LRRK2 kinase is constitutively activated, MLi-2 rescues DAT expression and function, and that treatment is paradoxically associated with AMPH-induced locomotion. Based on these results, we predict dopaminergic imaging of ^18^F-DOPA turnover^79^ in human *VPS35* p.D620N heterozygotes will inform target engagement and optimal dosing for LRRK2 kinase inhibitors that are now being advanced in clinical trials. Nevertheless, we caution that further pre-clinical studies are needed to dissect the cell-specific, pre- and post-synaptic roles of LRRK2 activity in dopaminergic circuits, and in the brain.

## Methods

### Animals

Vps35 constitutive knock-in (VKI) and haploinsufficient mice (Haplo) were concomitantly generated by Ozgene PLC (Australia), as previously described^17^. A floxed “mini-gene” consisting of: (1) splice acceptor, Vps35 exon 15–17 coding sequence, a polyadenylation signal (pA), and; (2) a PGK-neomycin -pA selection cassette (neo) internally flanked by FRT sites was inserted into intron 14. The 5′ targeting arm spanning endogenous exon 15 was used to introduce the Vps35 g.85,263,520 G > A (p.D620N) mutation (GRCm38/mm10; NM_022997.5). In this design, the mini-gene insertion inadvertently silenced the expression of the recombinant allele to create haploinsufficient mice (Haplo). Although Haplo heterozygotes are viable and fertile, they have ~50% lower levels of VPS35 compared to their wild type littermates. Double heterozygous crosses fail to breed to homozygosity as a complete loss of retromer is embryonic lethal^30^ (and unpublished data). Haplo animals crossed with transgenic mice expressing Cre recombinase successfully excised the floxed mini-gene cassette to produce animals that express VPS35 p.D620N (VKI). Both strains have been maintained on the same C57Bl/6 J background for >10 generations. Animal studies were approved by the Institutional Animal Care and Use Committee at University of Florida. All mice were kept on a reverse cycle (light on from 8:30 pm to 8:30am) and single-sex group-housed in enrichment cages after weaning at post-natal day 21, in accordance with NIH guidelines for care and use of animals. For all experiments, 3-6-month-old male animals were used. Ear notches were taken for DNA extraction and genotype validation.

### Gene and protein nomenclature

Gene nomenclature is consistent with published guidelines (https://useast.ensembl.org/index.html). Specifically, lower-case letters are given to mouse genes whereas upper case letters are used exclusively for human genes. Protein nomenclature is consistent with Uniprot. Specifically, upper case letters are used for both human and mouse protein, with the exception of Rab proteins, which appear as lower case on uniprot and many other publications.

### Perforated patch clamp electrophysiology

Mice were anesthetized with 5% isoflurane and then transcardially perfused with ice cold cutting solution containing (in mM): 205 sucrose, 2.5 KCl, 1.25 NaH_2_PO_4_, 7.5 MgCl_2_, 0.5 CaCl_2_, 10 glucose, 25 NaHCO_3_, and 1 kynurenic acid, saturated with 95% O_2_ and 5% CO_2_ (~305 mOsm/kg). Horizontal slices were prepared in the same cutting solution with a vibratome (Leica VT1200S) and incubated for 45 min-1 h at 32°C in a solution containing (in mM): 126 NaCl, 2.5 KCl, 1.2 NaH_2_PO_4_, 1.2 MgCl_2_, 2.4 CaCl_2_, 11 glucose, and 25 NaHCO_3_, saturated with 95% O_2_ and 5% CO_2_ (pH 7.4, ~300 mOsm/kg). Recordings were made at 33-34°C in the same solution perfused at 2 ml/min.

Perforated recordings were made using patch pipettes with an impedance of 3-7MΩ when filled with internal solution containing (in mM) 125 K-Gluconate, 4 NaCl, 10 Hepes, 4 Mg-ATP, 0.3 Na-GTP and 10 tris-phophocreatine (pH 7.3, ~280 mOsm/kg). Amphotericin was freshly diluted in internal solution at 195 nM concentration and used as a pore-forming agent for perforated recordings. Junction potentials were not corrected for all recordings. Cells were visualized using an upright microscope (Olympus OX50WI) with infrared/differential interference contrast optics at 40x magnification. Dopaminergic neurons were identified based on their spontaneous firing (1–5 Hz) with broad action potentials (Aps) > 1.5 ms and sag potential elicited by hyperpolarizing current injection (~-10 mV with -100 pA current injection).

### Fast scan cyclic voltammetry

Mice were sacrificed by rapid decapitation and 300 µm coronal slices containing the striatum were cut in ice-cold cutting solution (containing in mM: 130 NaCl, 10 glucose, 26 NaHCO_3_, 3 KCl, 5 MgCl_2_, 1.25 NaH_2_PO_4_, and 2 CaCl_2_), saturated with 95% O_2_ and 5% CO_2_ ( ~ 305 mOsm/kg, pH 7.2–7.4). Subsequently, the striatal slices were incubated at room temperature for >1 hr in an artificial cerebrospinal fluid (ACSF) (containing in mM: 130 NaCl, 10 glucose, 26 NaHCO_3_, 3 KCl, 1 MgCl_2_, 1.25 NaH_2_PO_4_, 2 CaCl_2_), saturated with 95% O_2_ and 5% CO_2_ (~300 mOsm/kg, pH 7.2–7.4). Recordings were made at 27–29°C in the same solution perfused at ~2.5 ml/min. Slices were visualized using an upright microscope (Olympus OX50WI) with infrared/differential interference contrast optics. Single pulse stimuli (150 µs duration) were delivered by nickel-chromium bipolar electrode (made in house) placed in the dorsolateral striatum isolated (A365, World Precision Instrument) and controlled with Clampex software. Electrically evoked dopamine responses were monitored using carbon fiber electrodes (Invilog, diameter: 32 µm, sensitivity: >20 nA/µM) placed ~100 µm from the stimulating electrode. Triangular waveforms (ramp from −400 mV to 1200 mV to −400 mV, 10 ms duration at 10 Hz) were used to detect the oxidation and redox peaks for dopamine between 700 and 800 mV and voltametric responses were recorded, standardized and analyzed with an Invilog Voltammetry system and software (Invilog Research Ltd., Finland). The input/output paradigm consisted of single pulses of increasing intensities (50–700 μA, delivered every 2 min/ 0.0083 Hz) to determine the input required to evoke ~70% of the maximum response, which was used for the rest of the experiment. Five single pulses were delivered at 0.0083 Hz to calculate an average dopamine transient to be used for a more accurate representation of the decay characteristics. At the end of each recording session, a 3-point calibration of each carbon fiber electrode was conducted (final concentrations: 0.5, 1.0 and 2.0 μM dopamine in ACSF).

### Amphetamine treatment and open field behavior

Amphetamine (d-Amphetamine Sulfate, A5880) was made fresh each day in sterile saline at 0.5 mg/ml. Individual animals were weighed and placed into an open field chamber (19 ×19 x 19 inch) and allowed to habituate for 30 min followed by subcutaneous injection of amphetamine at 2 mg/kg. Total distance traveled was recorded throughout habituation and for 60 min following amphetamine injection. Distance traveled was analyzed by custom-made matlab code.

### MLi-2 treatment

Mice were randomly assigned for MLi-2 (Tocris) or vehicle treatment (Captisol), delivered once daily via intraperitoneal (IP) injection for a total of 7 days. Mice were weighed daily before injection. A solution of 0.5 mg/mL MLi-2 in 45% Captisol was prepared for injection, with a vehicle control of 45% Captisol alone, and filter sterilized prior to use. Each mouse was weighed and injected with a dose of 5 mg/kg. The mice were sacrificed 1 hr after the final injection, and their brains were extracted for FSCV or immunoblotting.

### Immunoblotting

Striatal tissues were homogenized and lysed in 1x Lysis Buffer (Cell Signaling #9803 S) with 1 mM PMSF and 1X phosphatase inhibitors (Thermofisher; #36978 and #78427, respectively). Homogenates were spun at 12,000 g for 15 min at 4 °C and pelleted debris were removed. Samples were denatured with 5% 2-mercaptoethanol in 1x NuPage LDS sample buffer (#NP0008), and resolved by SDS-PAGE using NuPage 4–12% Bis–Tris gel (Thermo Fisher Scientific #NP0321BOX) and transferred to PVDF membranes (EMD Millipore). The blots were blocked for 1 h in 5% non-fat milk (or BSA for phosphoprotein) and incubated overnight with primary antibodies in blocking buffer at 4 °C. All antibody concentrations used are detailed in Supplementary Table 1. Following 3 × 15 min washes with TBS-T, the membranes were incubated at room temperature with secondary antibodies diluted in blocking solution for 1 h, washed 3 × 15 min and scanned using a Chemi-doc imaging system (Cell-Bio). All blots were processed in parallel and derive from the same experiments.

### Real-time PCR (rt-PCR)

RNA was isolated from mouse midbrain using the RNAqueous-Micro Kit RNA isolation (Thermofisher) and was reverse transcribed using a RETROscript reverse transcription kit (Thermofisher). rtPCR was performed and analyzed using an Applied Biosystems 7500 Real-Time PCR System Machine and software. Taqman gene expression assays for mouse DAT (Mm00438388_m1) and GAPDH (Mm99999915_g1) were assessed.

### Statistics and data reporting

All experiments, data processing and analysis were conducted in a blinded manner. All electrophysiology and fast-scan voltammetry trace were analyzed using clampfit (Molecular devices). Densitometric signal from western blots was analyzed in Image J software^80^ and normalized to either GAPDH loading control or non-phosphorylated total protein level. Data are presented throughout as mean ± SEM where “n” represents the number of slices or neurons, and “N” represents the number of animals. All data were tested for normality and statistics were performed using either nonparametric or parametric tests, as detailed in the figure legends, using Prism 8.4 (GraphPad).

### Reporting summary

Further information on research design is available in the Nature Research Reporting Summary linked to this article.

### Supplementary information

supplementary material

reporting summary

## Data Availability

The VKI and Haplo strains have been deposited in Jackson Labs (www.jax.org; *Vps35* knock-in: B6(Cg)-Vps35tm1.1Mjff/J; Stock No: 023409; floxΔneo Vps35; B6.Cg-Vps35tm1.2Mjff/J; Stock No: 021807) with open distribution supported by the Michael J Fox Foundation. The codes and scripts for analysis of the open field data are available at (https://github.com/Guenther0623/Argus). The datasets generated during the current study are available from the corresponding author and will be shared upon request.
