# Supplementary material for: Inhibition of LRRK2 kinase activity rescues deficits in striatal dopamine physiology in VPS35 p.D620N knock-in mice
Source: NPJ Parkinsons Dis. 2023 Dec 18;9:167. doi: 10.1038/s41531-023-00609-7 (PMC10728137; doi:10.1038/s41531-023-00609-7)
Supplement: Supplementary file 1 — supplementary material [file 41531_2023_609_MOESM1_ESM.pdf]

Supplementary Figure:

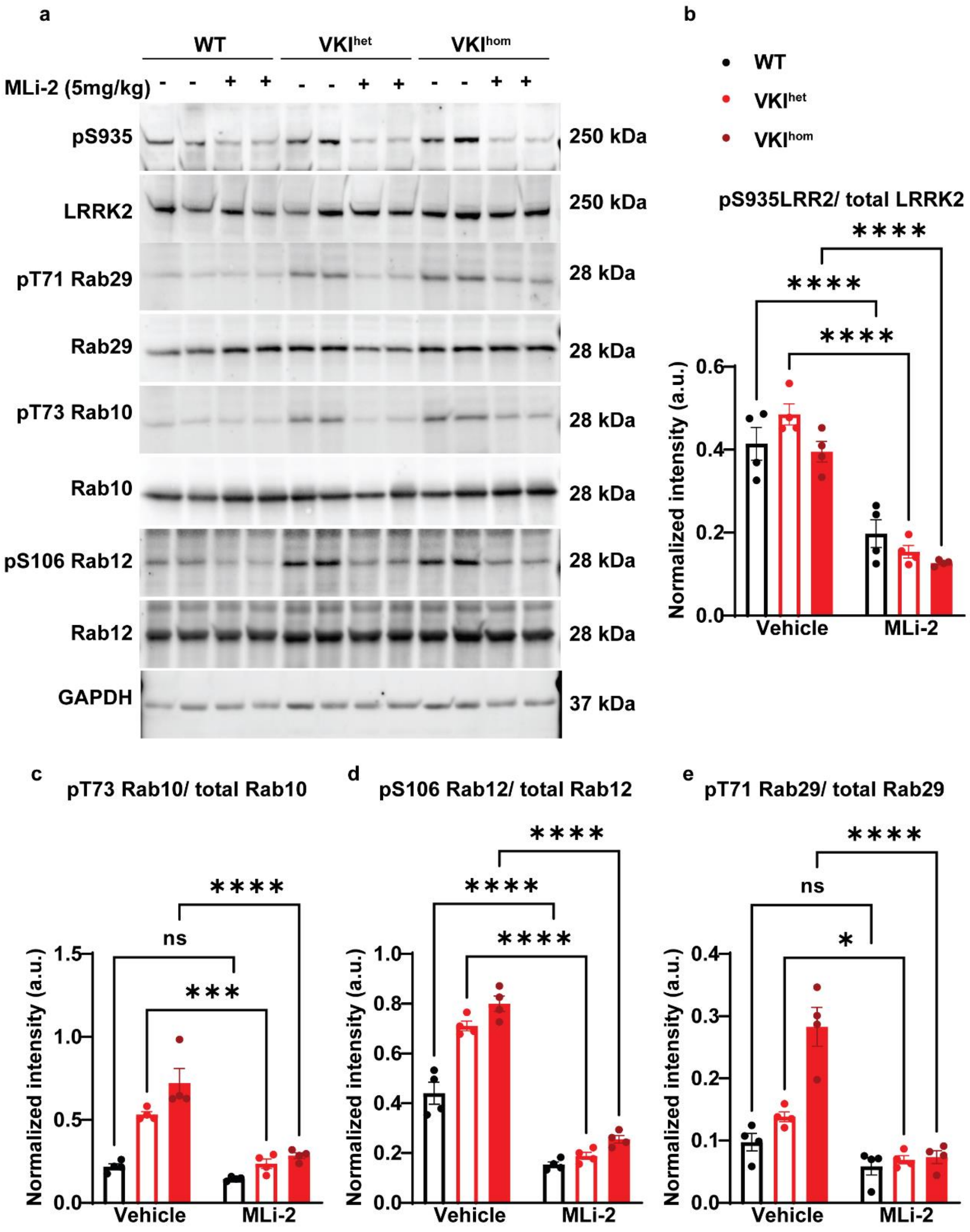

Supplementary Figure 1 (Related to Figure 2) 7-day *in-vivo* treatment by MLi-2 significantly reduced phospho-LRRK2 (pLRRK2) and phospho-Rabs in VKI. a: Representative blots of LRRK2 and Rab protein phosphorylation. **b**: MLi-2 treatment significantly reduced pS935-LRRK2 in all genotypes (**b**: N=4 for all groups; Two-way ANOVA followed by Sidak's multiple comparison: Genotype: $F_{2, 18}$ =2.69, p =0.09; Treatment: $F_{1, 18}$=157.9, p<0.0001 Genotype x Treatment: $F_{2, 18}$ = 2.35, p=0.12.) and downstream Rab10, 12 and 29 phosphorylation with a clear interaction between genotype and treatment. (**c-e**: N=4 for all groups; Two-way ANOVA followed by Sidak's multiple comparison: **c**: Genotype: $F_{2, 18}$ =33.48, p<0.0001; Treatment: $F_{1, 18}$=68.81, p<0.0001 Genotype x Treatment: $F_{2, 18}$ = 10.69, p=0.0009; **d**: Genotype: $F_{2, 18}$ =43.29, p<0.0001; Treatment: $F_{1, 18}$=479.3, p<0.0001 Genotype x Treatment: $F_{2, 18}$ = 15.96, p=0.0001; **e**: Genotype: $F_{2, 18}$ =20.89, p<0.0001; Treatment: $F_{1, 18}$=64.69, p<0.0001 Genotype x Treatment: $F_{2, 18}$ = 15.95, p=0.0001). *p < 0.05, **p < 0.01, ***p < 0.001, ****p < 0.0001. Error bars are ±SEM.s

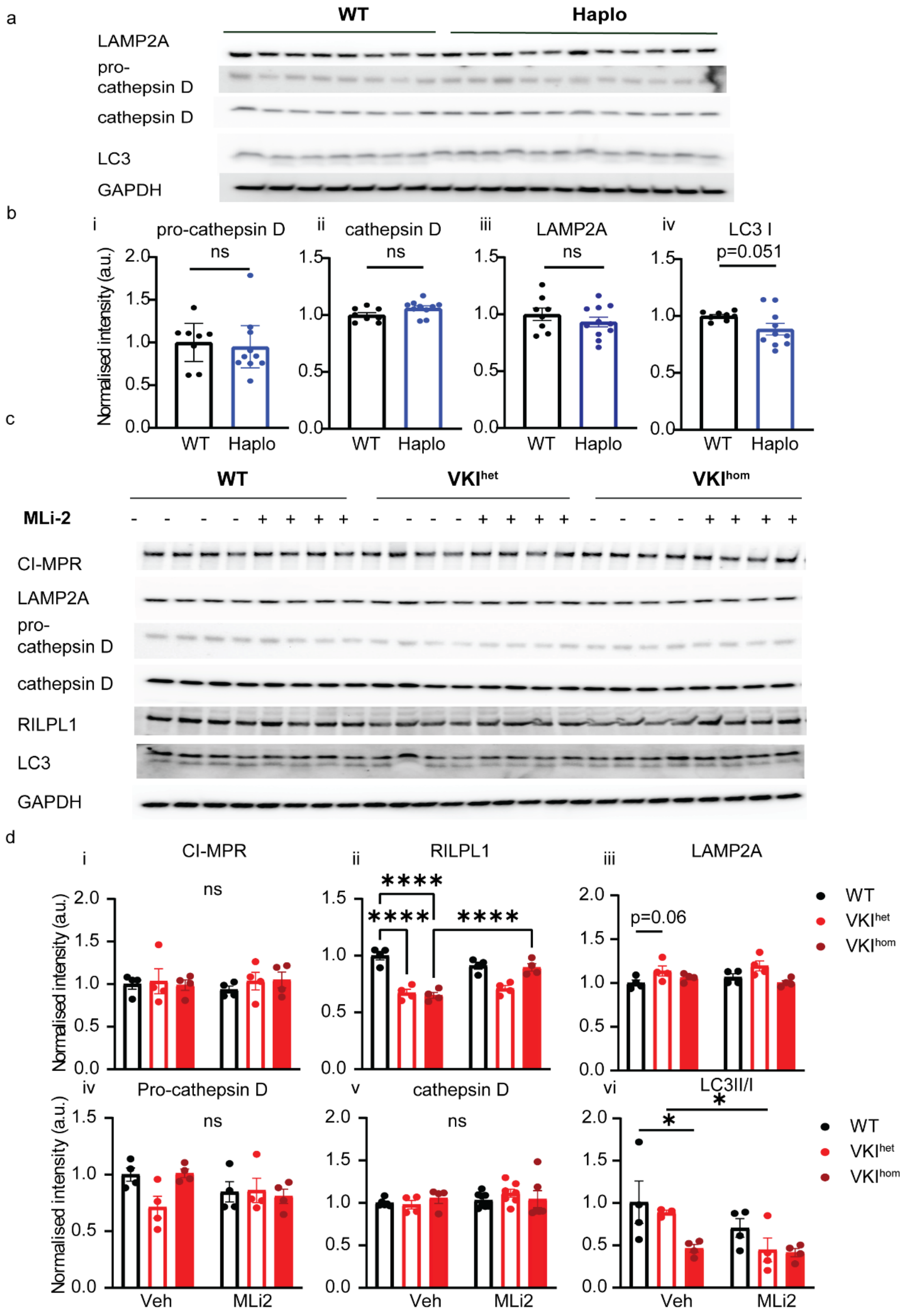
a
WT
Haplo
LAMP2A
pro-cathepsin D
cathepsin D
LC3
GAPDH
b
i pro-cathepsin D
ii cathepsin D
iii LAMP2A
iv LC3 I
ns
p=0.051
Normalised intensity (a.u.)
WT Haplo
c
WT
VKI[het]
VKI[hom]
MLi-2
CI-MPR
LAMP2A
pro-cathepsin D
cathepsin D
RILPL1
LC3
GAPDH
d
i CI-MPR
ii RILPL1
iii LAMP2A
iv Pro-cathepsin D
v cathepsin D
vi LC3II/I
ns
****
p=0.06
*
Normalised intensity (a.u.)
Veh MLi2
WT
VKI[het]
VKI[hom]

Supplementary Figure 2 (Related to Figure 2) Endo-lysosomal markers in Haplo and VKI animals: a, c: Representative blots of Haplo and VKI striatal homogenates**. b:** Quantification of pro- and mature-cathepsin D, LAMP2A and LC3 in Haplo mice. (**b-i-iv**: WT: N=8, Haplo N=11, unpaired t-test) **d**: Quantification of CI-MPR, RILPL1, LAM2A, pro- and mature-cathepsin and LC3 in VKI mice. (**d-i-v**: N=3-4 per group, Two-way ANOVA followed by Sidak's multiple comparison when significant; **(i)** Genotype: $F_{2, 18}$ =0.29, p>0.05; Treatment: $F_{1, 18}$=2e^{-6}, p>0.05; Genotype x Treatment: $F_{2, 18}$ = 0.23, p>0.05. **(ii)**Genotype: $F_{2, 18}$ =39.08, p<0.0001; Treatment: $F_{1, 18}$=6.73, p<0.05; Genotype x Treatment: $F_{2, 18}$ = 15.23, p<0.001. **(iii)** Genotype: $F_{2, 18}$ =6.94, p<0.01; Treatment: $F_{1, 18}$=0.47, p>0.05; Genotype x Treatment: $F_{2, 18}$ = 1.42, p>0.05. **(iv)** Genotype: $F_{2, 18}$ =1.81, p>0.05; Treatment: $F_{1, 18}$=1.42, p>0.05; Genotype x Treatment: $F_{2, 18}$ = 2.84, p>0.05. **(v)**Genotype: $F_{2, 18}$ =0.24, p>0.05; Treatment: $F_{1, 18}$=1.73, p>0.05; Genotype x Treatment: $F_{2, 18}$ = 0.79, p>0.05 **(vi)** Genotype: $F_{2, 18}$ =6.04, p<0.05; Treatment: $F_{1, 18}$=7.58, p<0.05; Genotype x Treatment: $F_{2, 18}$ = 1.43, p>0.05**)** *p < 0.05, **p < 0.01, ***p < 0.001, ****p < 0.0001. Error bars are ±SEM.s

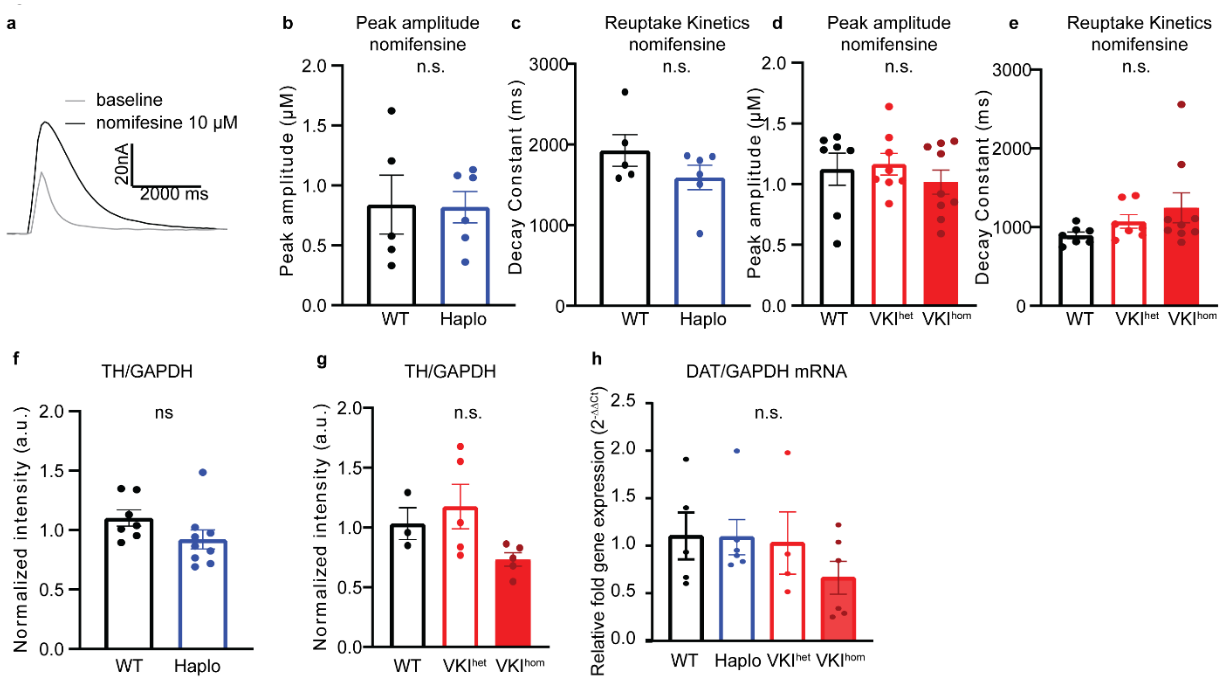

Supplementary Figure 3 (Related to Figure 4) Additional evaluation of striatal dopamine in Haplo and VKI animals: a: Representative traces of evoked dopamine release in the presence and absence of 10um nomifensine. **b-c**: Quantification of peak amplitude **(b)** and decay constant **(c)** with nomifensine treatment in Haplo (**b-c:** WT: n=5 slices N=5 animals; Haplo: n=6 slices N=6 animals; unpaired t test; **b:** t_9 = 0.081; **c:** t_9 = 1.38). **d-e**: Quantification of peak amplitude **(d)** and decay constant **(e)** with nomifensine treatment in VKI. (**d-e:** WT: n=7 slices N=5 animals; VKI^{het}: n=8 slices, N=5 animals; VKI^{hom} n=9 slices, N=6 animals; One-way ANOVA; **d**: $F_{2, 21}$ = 0.55; **e**: $F_{2, 23}$ = 1.95) **f-g**: Quantification of TH immunoblot blot (Fig. 4g) shows comparable striatal TH expression in Haplo (**f**: N=7 animals; Haplo: N=10 animals; unpaired t test: t_{14} = 1.65) and VKI animals (**g**: WT: N=3 animals; VKI^{het}: N=5 animals; VKI^{hom}: N=5 animals; One-way ANOVA: $F_{2, 10}$ = 2.92). **h:** rtPCR shows comparable level of DAT mRNA in both Haplo and VKI animals. (WT: N=5 animals; Haplo: N=6 animals; VKI^{het} : N=4 animals; VKI^{hom} : N=6 animals; One-way ANOVA; $F_{3, 17}$ = 0.96) Error bars are ±SEM.s

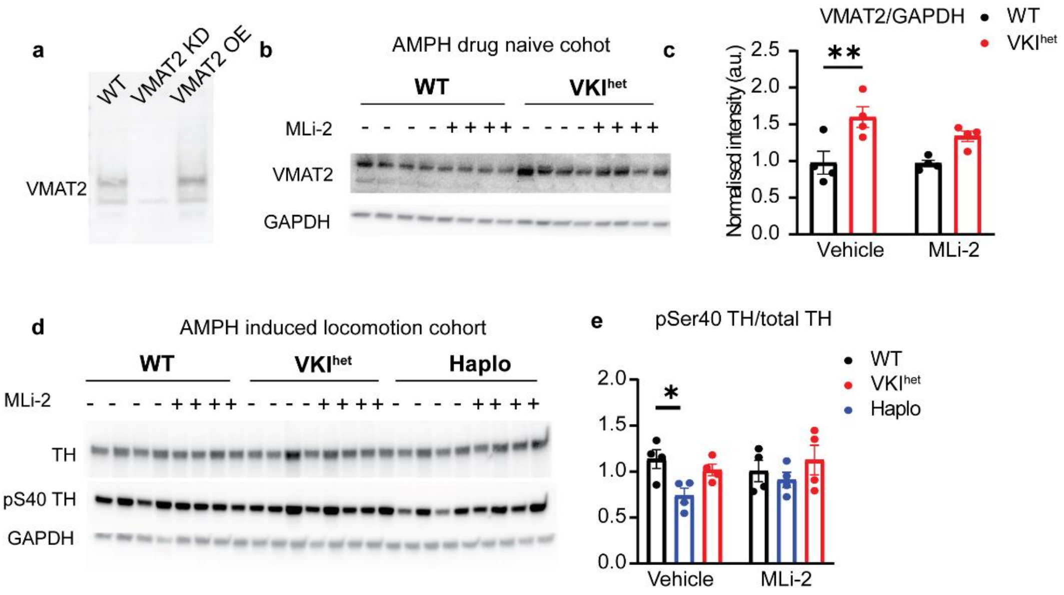

Supplementary Figure 4: (Related to figure 6) Dopaminergic markers in mice following 7-day MLi-2 and AMPH induced hyperlocomotion. a: VMAT2 antibody validation with midbrain homogenate from WT, VMAT knockdown, and VMAT2 overexpressing animals. **b:** Immunoblot of VMAT in striatal tissue homogenate of mice treated with MLi-2, without AMPH. **c**: VKI^{het} shows significant increase in VMAT2 expression at baseline (N=4 animals per group; Two-way ANOVA followed by Šídák's multiple comparisons test: Genotype: $F_{2,12} = 19.15$, $p<0.001$; Treatment $F_{1, 12}=1.43$; $p>0.05$; Interaction $F_{2, 12}= 1.12$, p>0..05). **d:** Immunoblots of dopaminergic markers of animals following MLi-2 and AMPH treatment. **e:** Vehicle treated Haplo animals exhibit significant reduction in phospho-serine40 TH levels compared to its WT counter part (N=4 animals per group; Two-way ANOVA followed by Šídák's multiple comparisons test: Genotype: $F_{(2, 18)} = 3.64$, $p<0.05$; Treatment $F_{1, 18}=0.33$; $p>0.05$; Interaction $F_{2, 18}= 1.12$, $p>0.05$)*$p < 0.05$, **$p < 0.01$, ***$p < 0.001$. Error bars are ±SEM.s

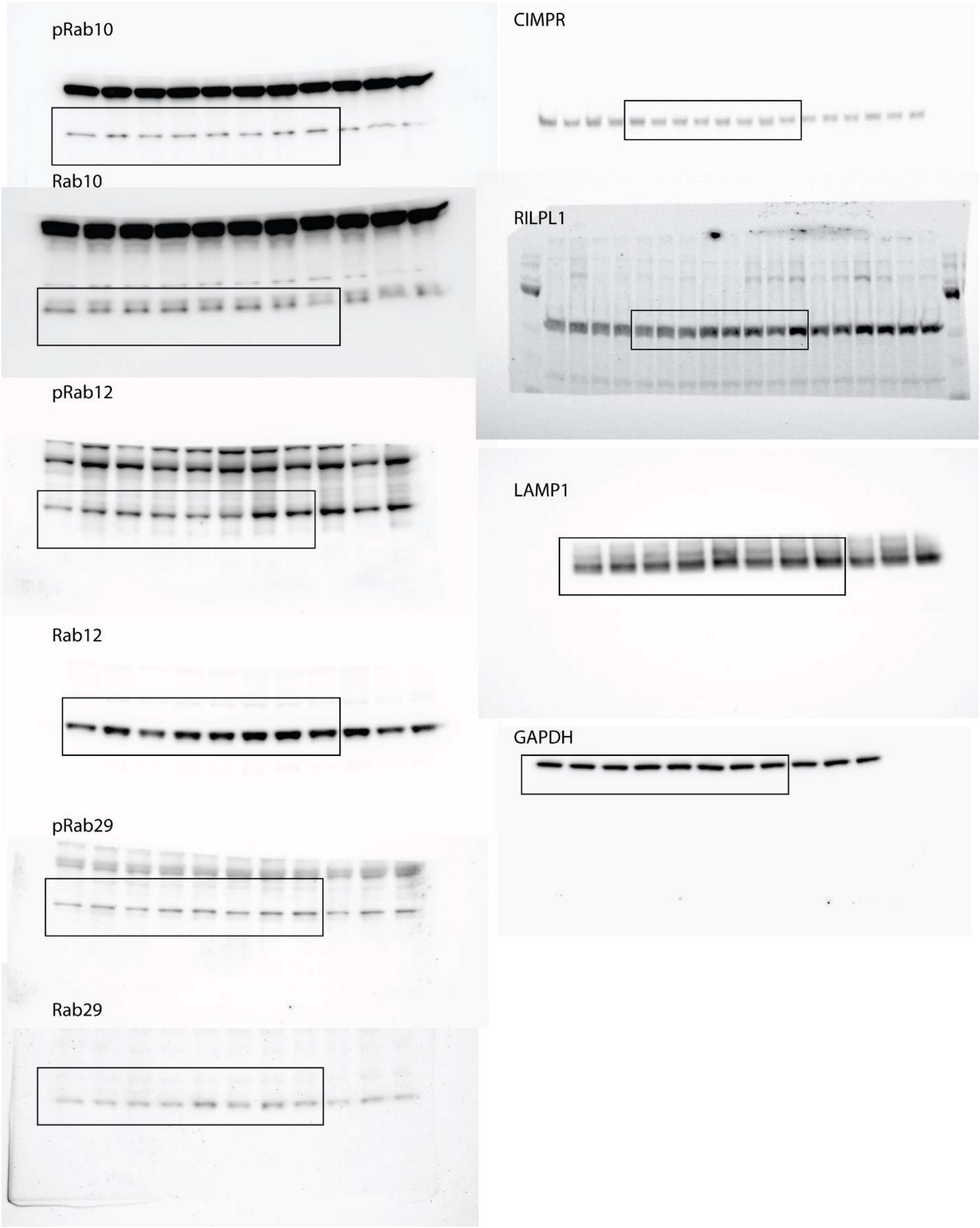

Supplementary Figure 5: Uncropped western blot shown in Fig.2a: Black boxes mark where western blots have been cropped for presentation

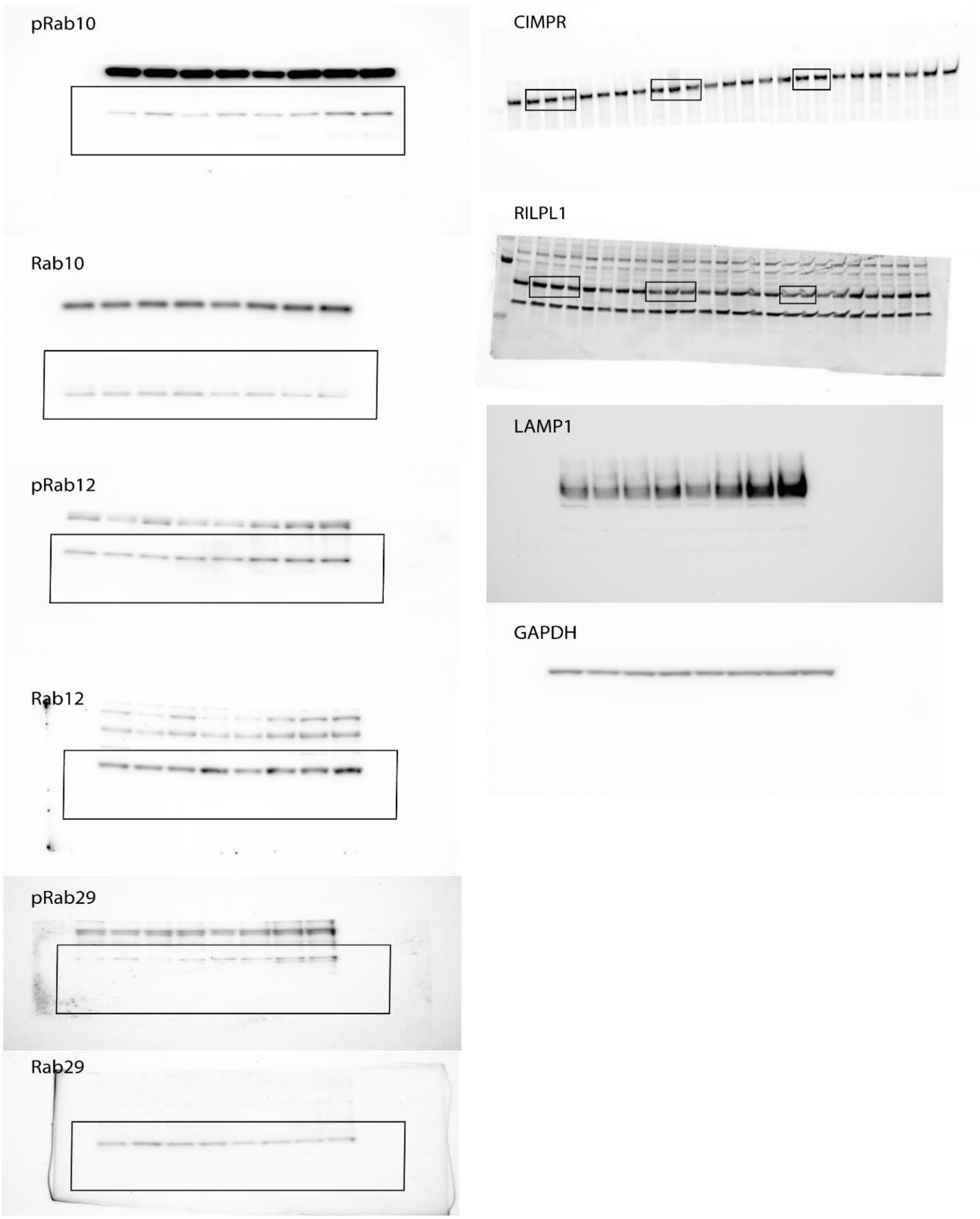

Supplementary Figure 6: Uncropped western blot shown in Fig.2b: Black boxes mark where western blots have been cropped for presentation. Note that uncropped version of RILP1 and CIMPR were also shown in FigS2

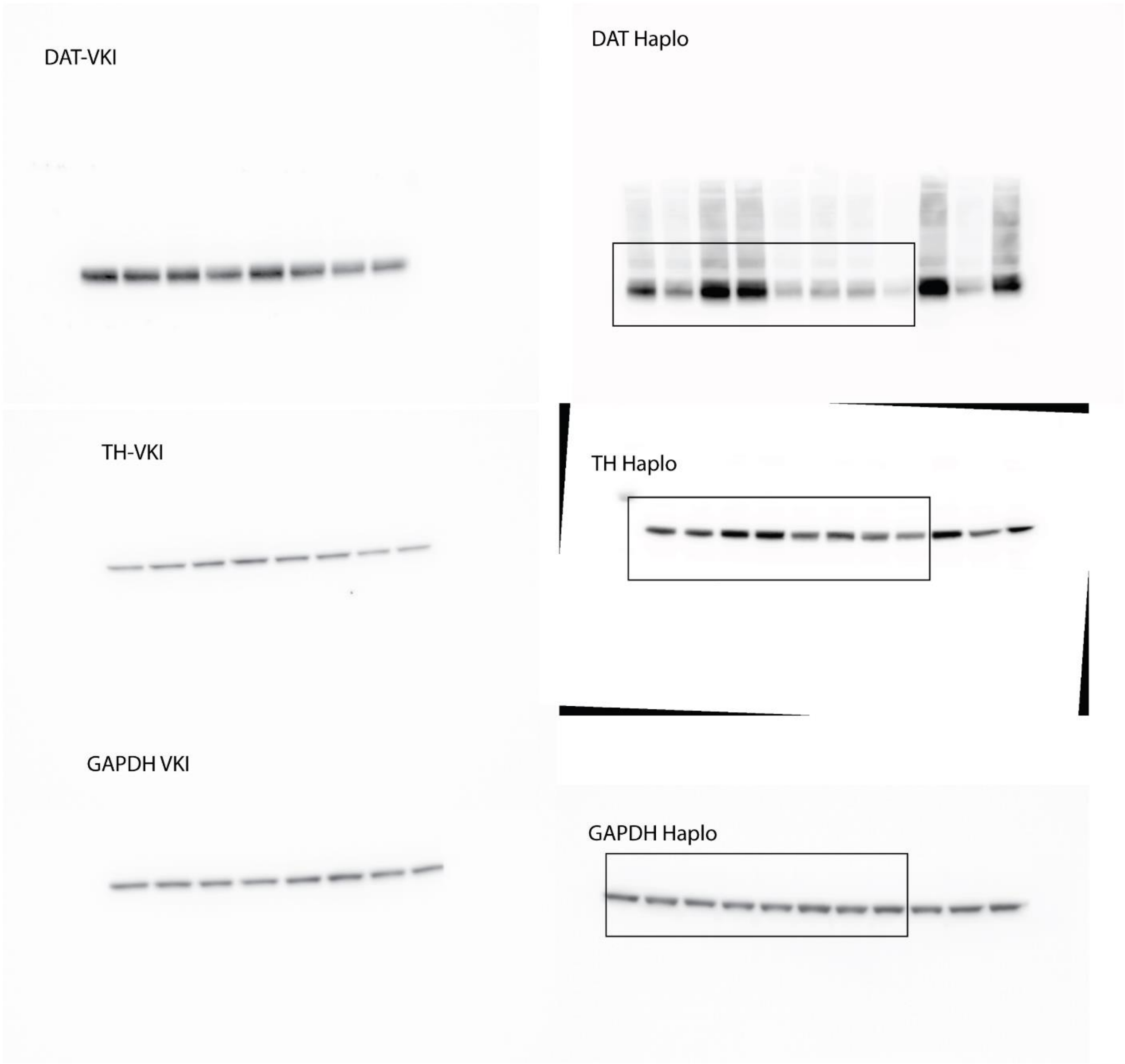

Supplementary Figure 7: Uncropped western blot shown in Fig.4g: Black boxes mark where western blots have been cropped for presentation.

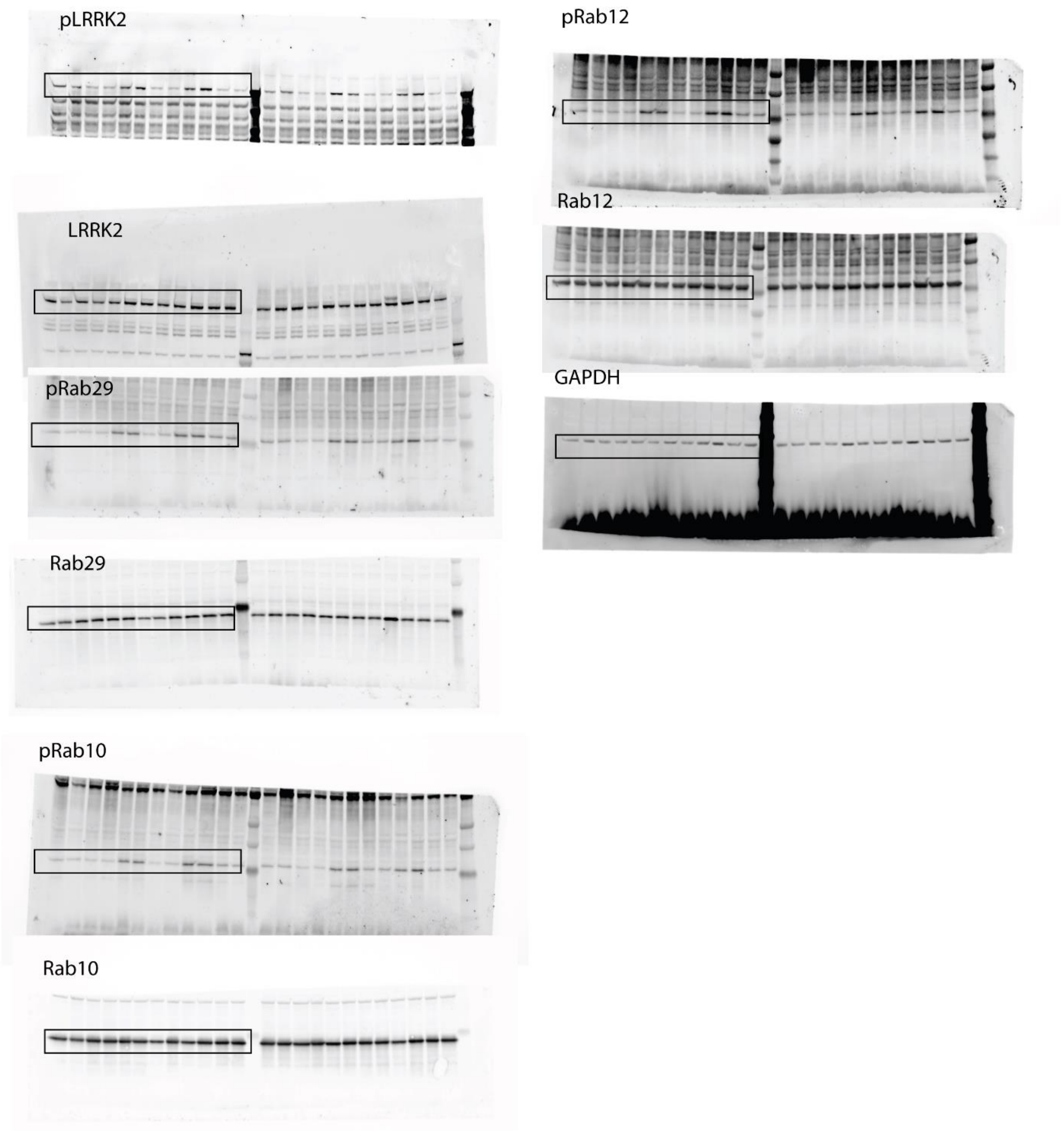

Supplementary Figure 8: Uncropped western blot shown in Supplementary Fig.1:

Black boxes mark where western blots have been cropped for presentation.

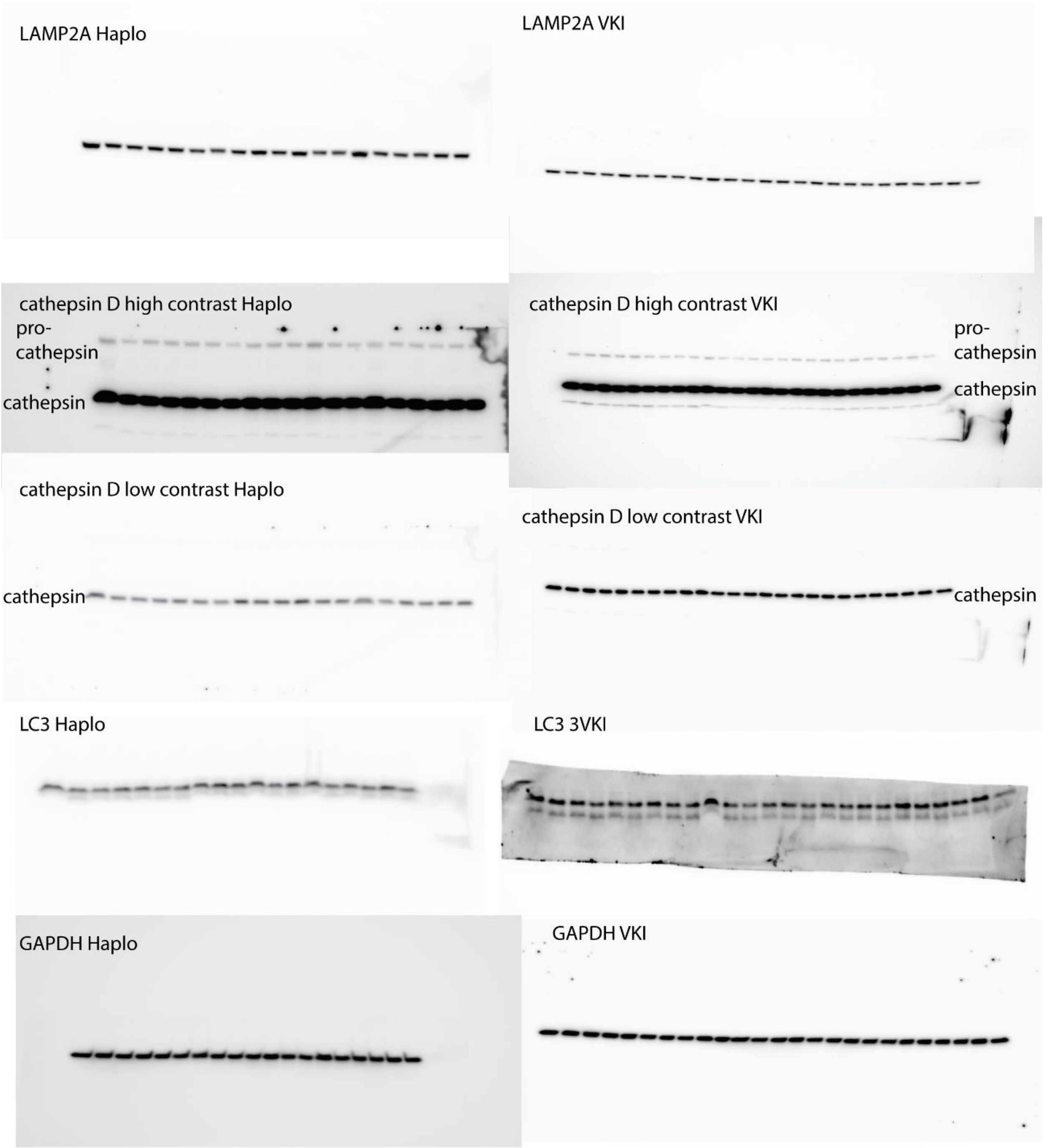

Supplementary Figure 9: Uncropped western blot shown in Supplementary Fig.2

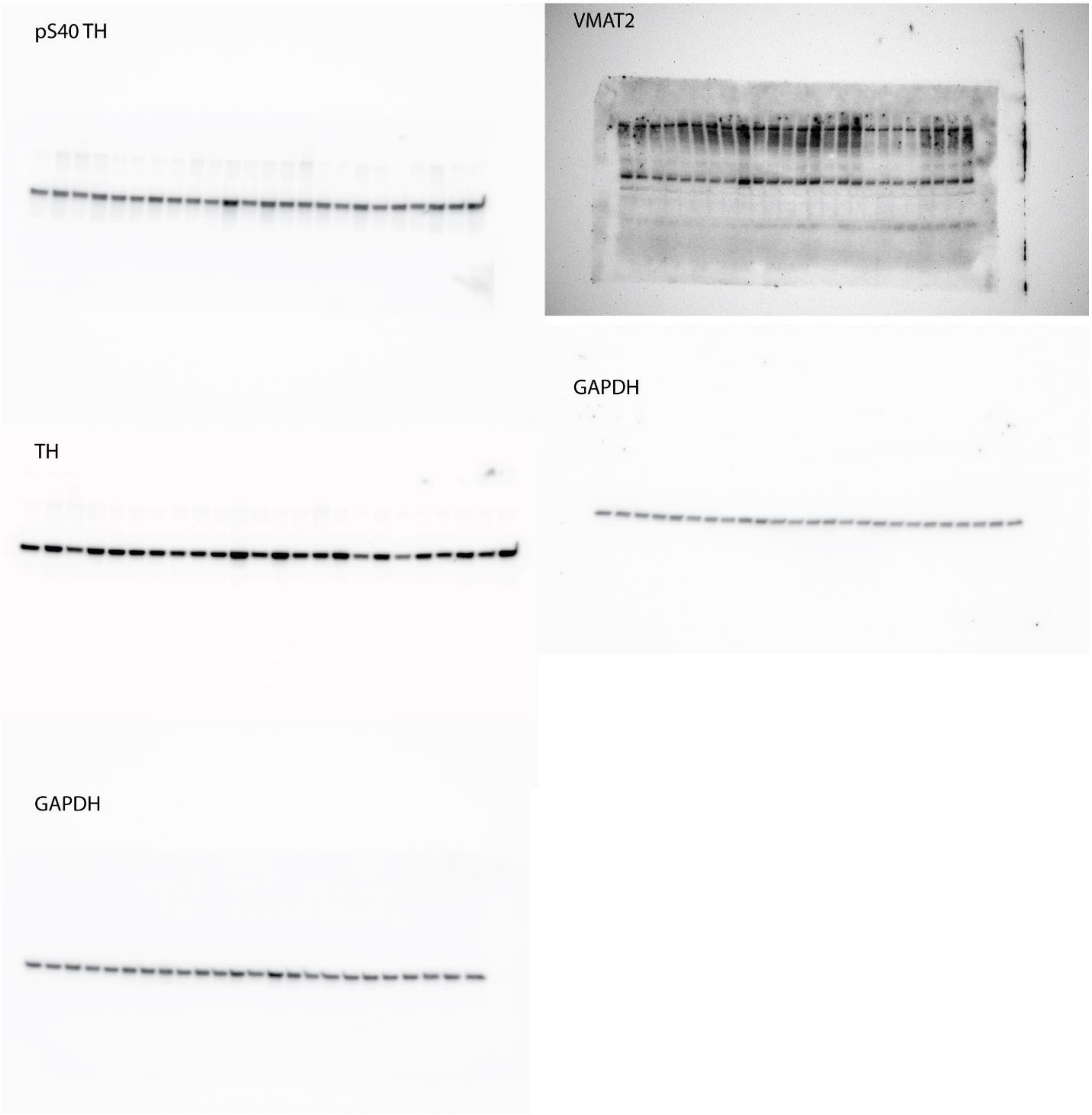

Supplementary Figure 10: Uncropped western blot shown in Supplementary Fig.4

Supplementary Table 1: List of antibodies and dilutions.

primary antibody	source	host species	Monoclonal/ Polyclonal	catalog number	primary concentration	secondary concentration
VPS35	Abnova	Mouse	Monoclonal	H00055737-M02	1:2000	1:2000
DAT	Millipore	Rat	Monoclonal	MAB369	1:1000	1:1000
TH	Millipore	Rabbit	Polyclonal	ab152	1:1000	1:1000
GAPDH	Thermo Scientific	Mouse	Monoclonal	MA5-15738	1:2000	1:2000
[MJF-R21] RAB10 (phospho T73)	Abcam	Rabbit	Monoclonal	ab230261	1:1000	1:1000
RAB10	Abcam	Rabbit	Monoclonal	ab237703	1:1000	1:1000
Rab12 pS106 (MJF-R25-9)	Abcam	Rabbit	Monoclonal	ab256487	1:1000	1:1000
RAB 12	Proteintech	Rabbit	Polyclonal	18843-1-AP	1:1000	1:1000
RAB29 (phospho T71) [MJF-R24-17-1]	Abcam	Rabbit	Polyclonal	ab241062	1:1000	1:1000
RAB29 [MJF-R30-124]	Abcam	Rabbit	Polyclonal	ab256526	1:1000	1:1000
LAMP1	Abcam	Rabbit	Monoclonal	ab208943	1:1000	1:1000
SQSTM1 (P62)	Abcam	Rabbit	Polyclonal	ab109012	1:1000	1:1000
LRRK2/Dardarin (IgG2a)	NeuroMab	Mouse	Monoclonal	N241A/34	1:500	1:500
LRRK2 p-S935	Abcam	Rabbit	Monoclonal	ab133450	1:500	1:500
VMAT2	Gift from lab of Dr. Gary Miller	Rabbit	Antisera	-	1 :2000	1:2000
RILPL1	Abcam	Rabbit	Monoclonal	AB302492	1:1000	1:1000
CI-MPR	Thermo Scientifc	Rabbit	Polyclonal	PA3-850	1:1000	1;1000
Cathepsin D	Abcam	Mouse	Monoclonal	ab6313	1:2000	1:2000
LC3	Abcam	Rabbit	Polyclonal	ab51520	1:1000	1:1000
mouse anti-rabbit IgG-HRP	Santa Cruz	Mouse	Polyclonal	sc-2357		
anti-mouse IgG-HRP	Santa Cruz		Polyclonal	sc-516102		
goat anti-rat IgG	biolegend	Goat	Polyclonal	poly4054		
